# Development of a bioinformatics platform for analysis of quantitative transcriptomics and proteomics data: the OMnalysis

**DOI:** 10.7717/peerj.12415

**Published:** 2021-11-09

**Authors:** Punit Tyagi, Mangesh Bhide

**Affiliations:** 1 Laboratory of Biomedical Microbiology and Immunology, University of Veterinary Medicine and Pharmacy in Kosice, Kosice, Slovakia; 2 Department of Animal and Food Science, The Autonomous University of Barcelona, Barcelona, Spain; 3 Institute of Neuroimmunology, Slovak Academy of Sciences, Bratislava, Slovakia

**Keywords:** Omics, Shiny, Transcriptomics, Proteomics, Bioinformatics tool, Functional profiling, RNA-seq, Data exploration

## Abstract

**Background:**

In the past decade, RNA sequencing and mass spectrometry based quantitative approaches are being used commonly to identify the differentially expressed biomarkers in different biological conditions. Data generated from these approaches come in different sizes (*e.g.*, count matrix, normalized list of differentially expressed biomarkers, etc.) and shapes (*e.g.*, sequences, spectral data, etc.). The list of differentially expressed biomarkers is used for functional interpretation and retrieve biological meaning, however, it requires moderate computational skills. Thus, researchers with no programming expertise find difficulty in data interpretation. Several bioinformatics tools are available to analyze such data; however, they are less flexible for performing the multiple steps of visualization and functional interpretation.

**Implementation:**

We developed an easy-to-use Shiny based web application (named as OMnalysis) that provides users with a single platform to analyze and visualize the differentially expressed data. The OMnalysis accepts the data in tabular form from edgeR, DESeq2, MaxQuant Perseus, R packages, and other similar software, which typically contains the list of differentially expressed genes or proteins, log of the fold change, log of the count per million, the *P* value, *q*-value, etc. The key features of the OMnalysis are multiple image type visualization and their dimension customization options, seven multiple hypothesis testing correction methods to get more significant gene ontology, network topology-based pathway analysis, and multiple databases support (KEGG, Reactome, PANTHER, biocarta, NCI-Nature Pathway Interaction Database PharmGKB and STRINGdb) for extensive pathway enrichment analysis. OMnalysis also fetches the literature information from PubMed to provide supportive evidence to the biomarkers identified in the analysis. In a nutshell, we present the OMnalysis as a well-organized user interface, supported by peer-reviewed R packages with updated databases for quick interpretation of the differential transcriptomics and proteomics data to biological meaning.

**Availability:**

The OMnalysis codes are entirely written in R language and freely available at https://github.com/Punit201016/OMnalysis. OMnalysis can also be accessed from - http://lbmi.uvlf.sk/omnalysis.html. OMnalysis is hosted on a Shiny server at https://omnalysis.shinyapps.io/OMnalysis/. The minimum system requirements are: 4 gigabytes of RAM, i3 processor (or equivalent). It is compatible with any operating system (windows, Linux or Mac). The OMnalysis is heavily tested on Chrome web browsers; thus, Chrome is the preferred browser. OMnalysis works on Firefox and Safari.

## Introduction

High-throughput techniques have emerged as powerful tools to study differential omics. RNA-sequencing (RNA-seq) and liquid chromatography coupled with mass spectrometry (LC-MS) based quantitative approaches are increasingly being used (Lagarrigue et al., 2013; Milanez-Almeida et al., 2020; Wang, Gerstein & Snyder, 2009). The low-cost data generation and available computational power have enabled the multi-omics studies in the past decade. Omics is composed of multiple layers (genomics, epigenomics, transcriptomics, proteomics, metabolomics, microbiomics, and phenomics), however, transcriptomics and proteomics remain the most commonly used omics (Gomez-Cabrero et al., 2014; Hasin, Seldin & Lusis, 2017; Yan et al., 2018). Transcriptomics and proteomics experiments require high-cost instrumental setup, labor-intensive sample preparation, and technical skills (Darville & Sokolowski, 2018; Hrdlickova, Toloue & Tian, 2017). As a result, researchers often outsource samples to core facilities and obtain log-transformed or normalized expression data, which require to be interpreted into biological relevance using bioinformatic tools. These tools are either scattered or lack updated algorithms or fail to use up-to-date annotated repositories, which are the prerequisites of the correct biological interpretation of the data (Mangul et al., 2019).

In the case of transcriptomics, the data-intensive preprocessing often delivers a data matrix (the output) that contains columns of identified genes, the magnitude of the change against control (*e.g.*, log fold change), significance value (*P* value), and transcript count (log counts per million). The output usually depends on the type of statistical package and function used, for example, EdgeR (Robinson, McCarthy & Smyth, 2010), DESeq2 (Love, Huber & Anders, 2014) and Cuffdiff (Trapnell et al., 2012). On the other hand, in the case of quantitative proteomics the data analyzed for quality check, peptide identification, protein quantification, and normalization (Darville & Sokolowski, 2018) deliver a table containing columns of identified proteins, the magnitude of change in abundance against control and FDR-adjusted *P*-value. Such data matrices can be analyzed using different bioinformatic tools depending on the study type and level of regulation. Myriad tools and web applications were developed to analyze the count data from the transcriptomics and list of the proteins from proteomics study. Some of them are iDEP (integrated differential expression and pathway analysis) (Ge, Son & Yao, 2018), IRIS (integrated RNA-seq data analysis and interpretation system) (Monier et al., 2019) and DEBrowser (Kucukural et al., 2019), which perform pre-processing, heatmaps construction, unsupervised learning, DEGs filtering, pathway analysis and submission to gene expression omnibus public repository. Such approaches, however, have limitations in addressing the user-friendliness, level of complication, input data requirements, publication-ready visualization (image formats), multiple database coverage, and access to the published research article.

In omics studies, it is often difficult to narrow down 2–5 top biomarkers from thousands of differentially expressed genes (DEGs) or protein (DEPs). It requires moderate computational effort, iterative re-testing, and use of the multiple databases. Towards this end, we developed an R Shiny based web-tool—the OMnalysis, that accepts the list of transcriptomics and proteomics normalized quantitative expression data generated from bioinformatics tools like edgeR version 3.32.0 (Robinson, McCarthy & Smyth, 2010) and Perseus version 1.6.15.0 (Tyanova et al., 2016). Using the flexdashboard with R Shiny, we designed the flexible and easy-to-use platform, that integrates the highly reviewed R packages. Shiny based applications are known for the ease to build an interactive web application from R, providing higher flexibility, integration of other programming languages (JavaScript actions, Html, CSS), and standalone hosting on the webpage. The OMnalysis includes real-time accession ID conversion using: biomaRt version 2.46.3 (Durinck et al., 2009). It also enables multiple visualization options with customization of the resolution, dimension, and image format. In addition, it is also supported by KEGG, Reactome, biocarta, PANTHER, nature pathway interaction database (NCI), pharmGKB, and STRING to perform in-depth significant enrichment analysis and increase the annotation coverage of the input set of genes and proteins. Furthermore, it can analyze up to four treatments simultaneously with no additional requirement of metadata. Using Europe PMC (Levchenko et al., 2018), this tool also provides access to the millions of published scientific literature to acquire relevant information on the biomarkers analyzed.

## Material and Methods

### Software packages and implementation

OMnalysis is an interactive R Shiny based web application composed of multiple sectioned user interface (UI) in the form of tabs. It is built to perform exploration of differential expression data efficiently and iteratively. R packages used for the development of the UI and its components are as follows: R Shiny version 1.6.0 (Chang et al., 2017), flexdashboard version 0.5.2 (Iannone, Allaire & Borges, 2018), Shiny Themes version 1.2.0 (Chang et al., 2018), rmarkdown version 2.8 (Allaire et al., 2020), knitr version 1.33 (Xie, 2019) and Shiny dashboard version 0.7.1 (Chang, Ribeiro & Barbara, 2019). Each sectioned UI is further divided into interactive and display panels. The interactive panel works on the Shiny’s reactivity property, which automatically updates the values in the output panel when the user interacts or changes the input components (plots, tables, actions, etc.). The biological analysis is supported by the following R packages: biomaRt version 2.46.3 (Durinck et al., 2009), clusterProfiler version 3.18.1 (Yu et al., 2012), reactomePA version 1.34.0 (Yu & He, 2016), reactome.db version 1.74.0 (Ligtenberg, 2019), pathview version 1.30.1(Luo & Brouwer, 2013), SPIA version 2.42.0 (Tarca et al., 2009), SBGNview version 1.4.1 (Dong et al., 2021), STRINGdb version 2.2.2 (Szklarczyk et al., 2019), org.Hs.eg.db, org.Gg.eg.db, org.Ss.eg.db, org.Bt.eg.db, (org.Mm.eg.db), (org.Rn.eg.db), (org.Cf.eg.db), (org.Dm.eg.db) and (org.Ce.eg.db)version 3.12.0 (Carlson, 2019). Visualization of the results from the analysis is backed by the following packages: EnhancedVolcano version 1.8.0 (Blighe, Rana & Lewis, 2020), gplots version 3.1.1 (Warnes et al., 2016), ggbiplot version 0.55 (Vu, 2016), ggplot2 version 3.3.3 (Wickham, Chang & Wickham, 2016), VennDiagram version 1.6.20 (Chen & Boutros, 2011), wordcloud version 2.6 (Fellows, 2018), dplyr version 1.0.5 (Wickham et al., 2015) and DT version 0.18 (Xie, Cheng & Tan, 2018).

**Figure 1:**
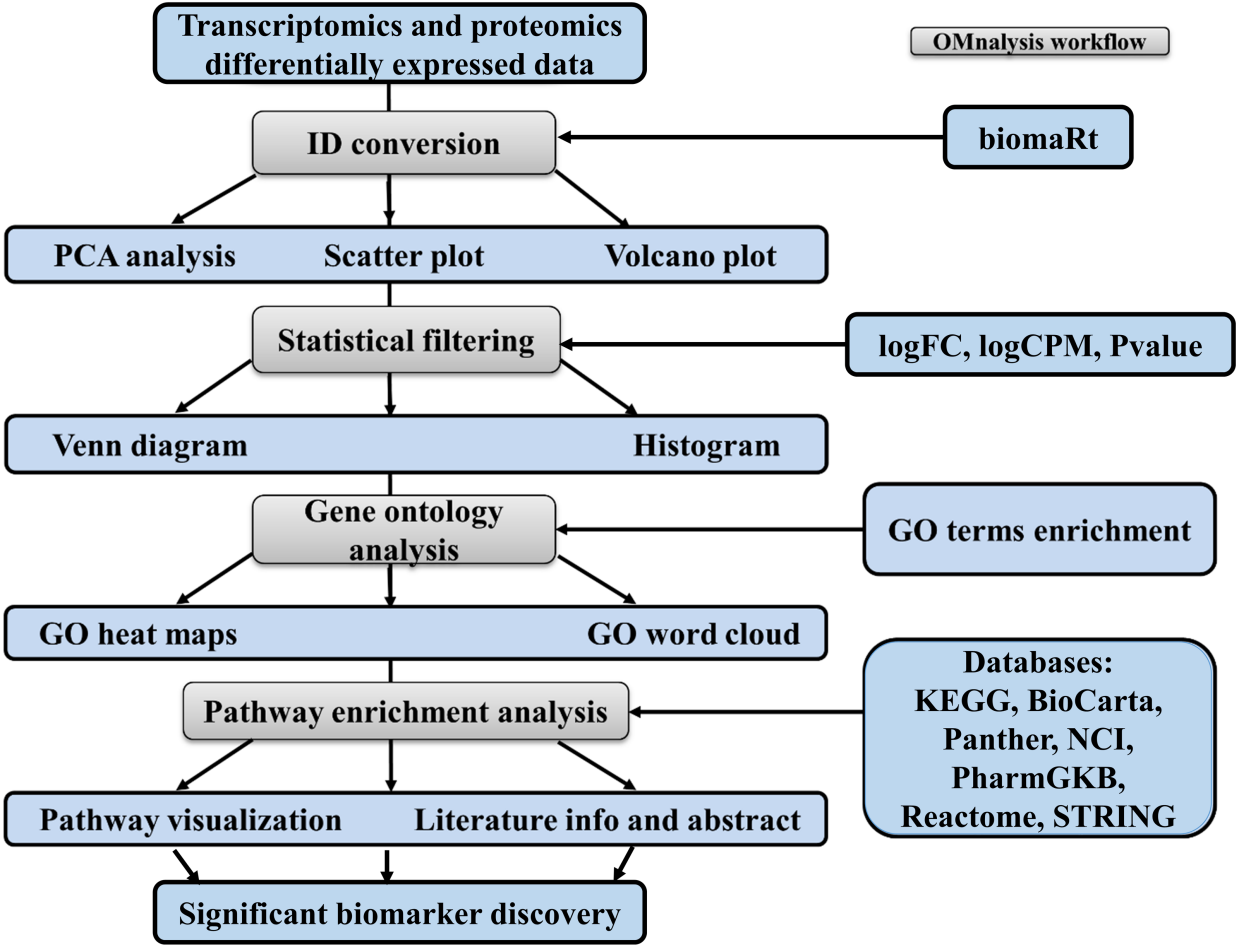
Workflow of OMnalysis web application. The OMnalysis pipeline starts with the DEGs or DEPs, allowing ID conversion, statistical filtering, functional annotation, pathway analysis, literature search, and finally provides information for the selection of significant biomarkers.

To enhance the user experience and proper execution of the OMnalysis pipeline (Fig. 1), peer-reviewed R packages were streamlined in multi-tabbed UI as follows: *Upload data, PCA, Plots, Statistical filtering, GO enrichment analysis, GO heatmaps, Pathway enrichment analysis, Enriched pathway visualization, Literature info and Help*.

The web application is designed to analyze two types of quantitative omics, the transcriptomics and proteomics. For transcriptomics, RNA-Seq data generated previously by us and deposited in ArrayExpress was used (http://www.ebi.ac.uk/arrayexpress). Expression analysis was performed on human brain microvascular endothelial cells (hBMEC) induced with various pathogens: *Borrelia burgdorferi* (Treatment1, Table S1, retrieved from ArrayExpress accession number E-MTAB-8053), *Neisseria meningitidis* (Treatment2, Table S2, E-MTAB-8008), *Streptococcus pneumoniae* (Treatment3, Table S3, E-MTAB-8054), and West Nile Virus (Treatment4, Table S4, E-MTAB-8052). Table S1 to Table S4 are in text format processed from the TSV file generated from the edgeR’s **glmTreat** function (Robinson, McCarthy & Smyth, 2010). Three columns (logFC, logCPM, and *P* value) from each of those supplemental files were copied to make a master file in CSV format (Table S5). For the sake of simplicity, Table S5 is explained in Table 1.

**Table 1:** Representative part of the input data for OMnalysis. The table presents part of Supplemental file 5. The underlined part in the table explains the source and it is not a part of the data matrix. Please note that the input table must contain the following headers: ENSEMBLGENE, logFC, logCPM and *P* value. The first column must be Ensembl IDs and then logFC, logCPM and Pvalue of each treatment in order as shown in table. col –column. In Pvalue column, user can add either *P* value or FDR adjusted *P* value.

Ensembl IDs	Treatment1			Treatment2			Treatment3			Treatment4		
**col. 1**	**col. 2**	**col. 3**	**col. 4**	**col. 5**	**col. 6**	**col. 7**	**col. 8**	**col. 9**	**col. 10**	**col. 11**	**col. 12**	**col. 13**
ENSEMBLGENE	logFC	logCPM	Pvalue	logFC	logCPM	Pvalue	logFC	logCPM	Pvalue	logFC	logCPM	Pvalue
ENSG00000000003	−0.74375	4.557846	0.267133	−0.90616	4.557846	0.141092	0.417361	4.557846	0.581714	−0.35996	4.557846	0.640167
ENSG00000000419	0.108453	4.758842	0.890174	0.096469	4.758842	0.902259	−0.12866	4.758842	0.872534	−0.78139	4.758842	0.254466
ENSG00000000460	−0.45651	1.782839	0.559013	−0.13903	1.782839	0.8607	−1.43246	1.782839	0.043123	0.792963	1.782839	0.277646
ENSG00000000971	−0.45012	6.933399	0.553369	−0.32462	6.933399	0.676356	0.131856	6.933399	0.868554	−0.62059	6.933399	0.380078
ENSG00000001036	−0.06613	5.462408	0.934619	−0.35845	5.462408	0.642259	−0.29022	5.462408	0.710371	0.171774	5.462408	0.827248
ENSG00000001084	−0.18668	4.100634	0.813381	−0.62329	4.100634	0.381325	0.057774	4.100634	0.941401	−0.01379	4.100634	0.986338
ENSG00000001167	0.414895	3.653166	0.584106	0.34253	3.653166	0.656407	−0.38414	3.653166	0.615672	0.178518	3.653166	0.820939
ENSG00000001461	−0.73107	5.259942	0.275607	−1.37751	5.259942	0.002377	−0.39492	5.259942	0.606211	−1.1324	5.259942	0.0298
ENSG00000001497	0.018868	4.674989	0.980772	0.013774	4.674989	0.985861	−0.41221	4.674989	0.589126	−0.54674	4.674989	0.454072
ENSG00000000003	−0.74375	4.557846	0.267133	−0.90616	4.557846	0.141092	0.417361	4.557846	0.581714	−0.35996	4.557846	0.640167

In the case of proteomics, the data matrix was generated from one of the differential abundance analysis software Perseus version 1.6.15 (Tyanova et al., 2016). To check the functionality, we retrieved the .xlsx format file from the experiment performed to quantify protein abundance in the milk whey collected at different time points from the cow with *Streptococcus uberis* infection (Mudaliar et al., 2016). The columns in this data matrix were arranged in the following order: UniProt ID, FDR-adjusted *P*-value, and Fold Change in an excel file for each experimental condition (4 time points in this case, designated as Treatment1, Treatment2, Treatment3, and Treatment4; Table S6).

### Data modification and ID conversion

We used the **read.csv** function of Utils package version 3.6.2 (Team, 2013) to upload the CSV format file (Table S5) to the OMnalysis using a tab “differentially expressed example data”. Whereas, for proteomics, we used the **import_list** function of rio package version 0.5.26 (Chan et al., 2018) to upload the data (Table S6) using a tab “proteomics abundance example data”. For proteomics data, three functions were used to convert data from Table S6 to make input table for the OMnalysis. First, the rio package was used to convert Treatment sheets to Treatment column. Second, the boldlog2 function of base R was used to transform the Fold Change column values to logFC (log Fold Change), and the third, **Colnames** function was used to change the treatment column name to Treatments. The duplicate proteins in the treatments were identified using the **group_by** function of dplyr version 1.0.5 (Wickham et al., 2015) and the **mean** function of base R to obtain the mean of their log fold change value. Such conversion is not necessary in case of transcriptomic data.

Transcriptomic data matrix contains Ensembl IDs, while proteomic data comes with UniProt IDs. To convert these IDs into five different ID types (Ensemble gene ID, gene name, HGNC symbol, gene description, and UniProtKB/Swiss-Prot ID) we used the **getBM** function of biomaRt package version 2.46.3 (Durinck et al., 2009) to fetch the latest information from the Ensembl database (Yates et al., 2020). We have incorporated the possibility of ID conversion for 9 species (Human, Chicken, Pig, Cow, Mouse, Rat, Dog, *Drosophilla melanogaster and C.elegans*) in OMnalysis.

### Principle component analysis (PCA)

**Figure 2:**
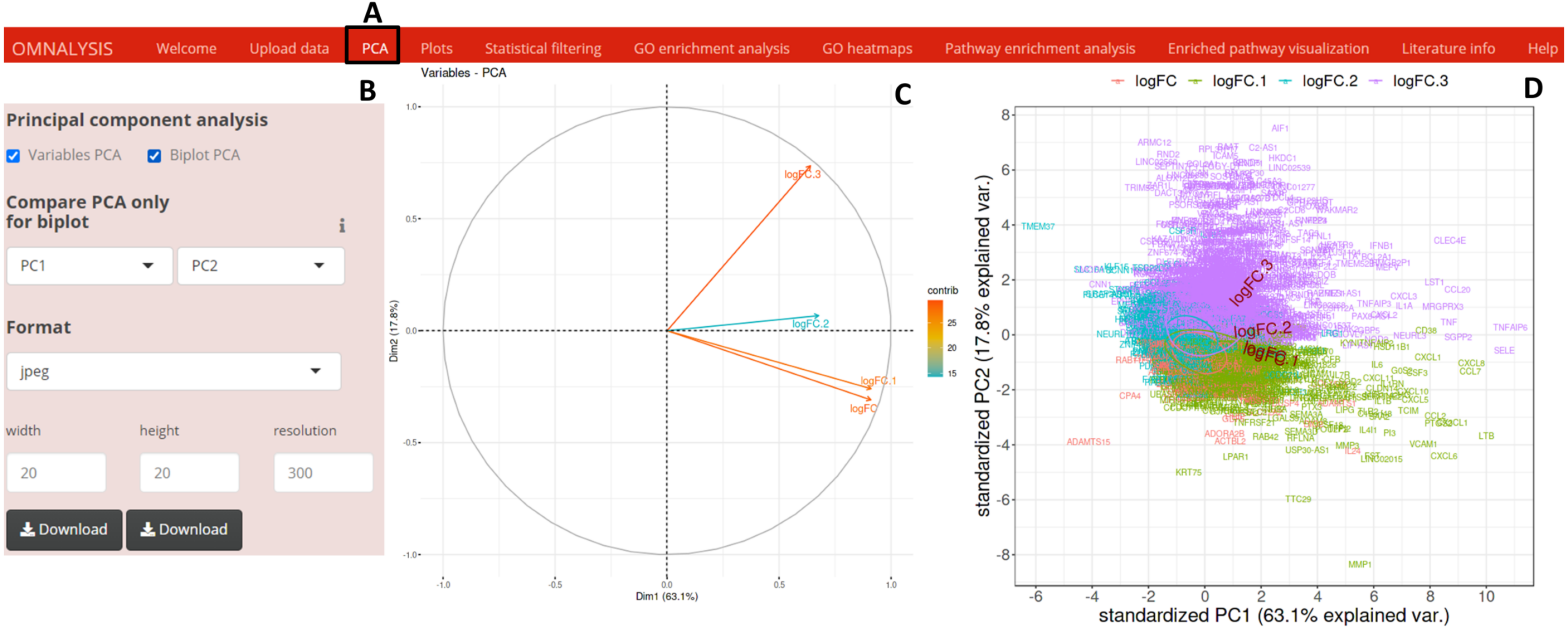
PCA and visualization using OMnalysis. (A) The user interface for PCA. (B) An interactive panel of OMnalysis to generate PCA plots and download them. (C) A variable PCA plot that shows the variability and contribution of genes among treatments. Lesser is the angle between two arrows higher is the positive correlation. (D) Biplot PCA presenting biomarkers and variability using the first two principal components.

To perform PCA, following the data matrix upload, the UI *PCA* tab (Fig. 2A) was included. In the interactive panel (Fig. 2B) for PCA, checkboxes and dropdown menu were inserted for Variable PCA and Biplot PCA. The **fviz_pca_var** function of factoextra package version 1.0.7 (Kassambara & Mundt, 2017) and **prcomp** function of stat version 3.6.2 (Team, 2013) were used for Variable PCA and Biplot PCA, respectively. The **biplot** function of ggbiplot version 0.55 (Vu, 2016) was used for the visualization (example data is shown in Figs. 2C and 2D).

### Plots

**Figure 3:**
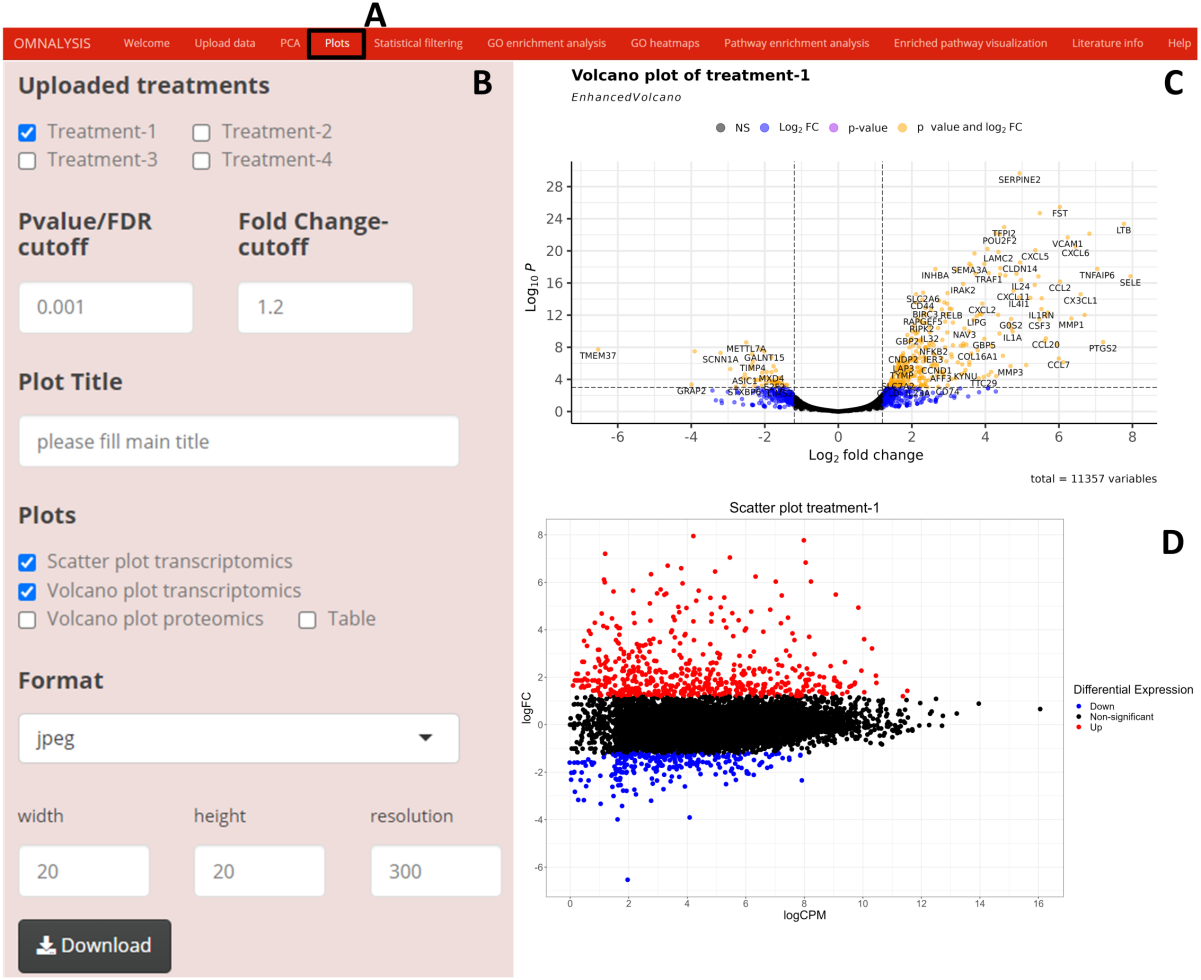
Generation of plots using OMnalysis. (A) The user interface for the plot. (B) An interactive panel to provide the input required to generate plots, customizable download dimensions, and image formats. (C) An example of a volcano plot with significantly up and down regulated genes (orange dot presents genes). (D) Example of the scatter plot providing information about differential expression using log fold change *vs.* log count per million.

We have created UI tab *Plots* (Fig. 3A) for generation of the scatter and volcano plots. An interactive panel (Fig. 3B) was created using flexdashboard version 0.5.2 (Iannone, Allaire & Borges, 2018), which accommodates the following options to users: selection checkboxes to compare the treatments and numeric input boxes to add values for *P* value and log fold changes (*P* value and FC-cutoff in Fig. 3B). We also introduced three checkboxes in the interactive panel for Scatter and Volcano plot for transcriptomics, whereas, Volcano plot checkbox for proteomics. Multiple image format drop-down menu and dimension correction numeric input options were also included to customize the generated plot (Fig. 3B). EnhancedVolcano package version 1.8.0 (Blighe, Rana & Lewis, 2020) and ggplot2 package version 3.3.3 (Wickham, Chang & Wickham, 2016) were used to generate an example volcano plot (Fig. 3C) and scatter plot (Fig. 3D) from DEGs or DEPs, respectively.

### Statistical filtering

**Figure 4:**
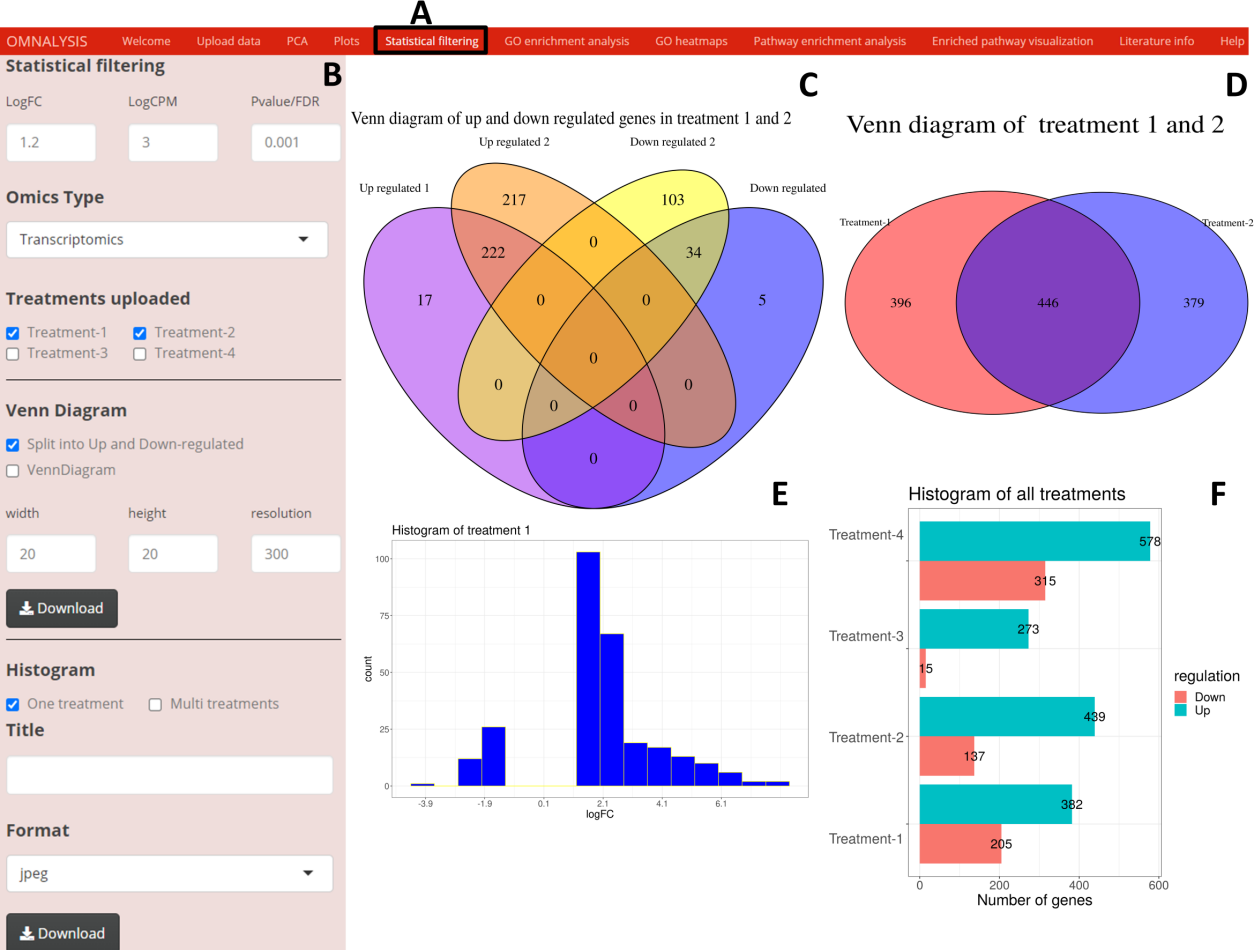
An overview of visualization after statistical filtering. (A) The user interface for Statistical filtering and diagrams. (B) An interactive panel for user input, treatment selection, omics selection, visualization type (Venn diagram or histogram), diagram customization, and download. (C) Split Venn diagram of up and down regulated common and unique genes in treatments 1 and 2. (D) Present a Venn diagram that shows the total number of differentially expressed genes in two treatments regardless of their up or down regulation. (E) A histogram providing a range of up and down regulation plotted against the number of the genes. (F) A histogram overview of the up and down regulated genes in all treatments after statistical filtering.

Following the PCA and plots, we included the *Statistical filtering* option in the UI tab (Fig. 4A). The interactive panel in this UI was populated with checkboxes to select treatments (where user can select the treatments for filtering and comparison). A dropdown menu “Omics Type” was added to select transcriptomics or proteomics. A numeric input box “Statistical filtering” was added to insert values for cutoff. Various checkboxes under the Venn Diagram and Histogram were added to plot the graphs based on cutoff values (Fig. 4B). The VennDiagram package version 1.6.20 (Chen & Boutros, 2011) and ggplot2 package version 3.3.3 (Wickham, Chang & Wickham, 2016) were used to plot Venn diagram (Figs. 4C and 4D) and histogram (Figs. 4E and 4F), respectively.

### Gene ontology analysis

Once the data matrix was statistically filtered, we used clusterProfiler version 3.18.1 of Bioconductor packages (Yu et al., 2012) to obtain the functional interpretation of the significantly expressed genes or proteins. Two enrichment analysis functions of the clusterProfiler were used, the first, **EnrichGO** function on genes or proteins to perform over representation analysis (ORA) and the second **gseGO** function on sorted genes or proteins with respect to logFC values to perform gene set enrichment analysis (GSEA) (Fig. S1). To support the enrichment analysis, AnnotationDbi version 1.52.0 of Bioconductor databases (Pagès et al., 2020) was used. Mark Carlson species-specific genome-wide annotation databases version 3.12.0 was used for human (org.Hs.eg.db), chicken (org.Gg.eg.db), pig (org.Ss.eg.db), and cattle (org.Bt.eg.db), mouse (org.Mm.eg.db), rat (org.Rn.eg.db), dog (org.Cf.eg.db), *Drosophila melanogaster* (org.Dm.eg.db) and *C.elegans* (org.Ce.eg.db) (Carlson, 2019). *P*-value cutoff input and **PAdjust** function of ClusterProfiler with seven multiple hypotheses testing correction methods ( Fig. S1) were used to avoid the influence of false-positive results on the overall enrichment analysis.

### Heatmap and word cloud

**Figure 5:**
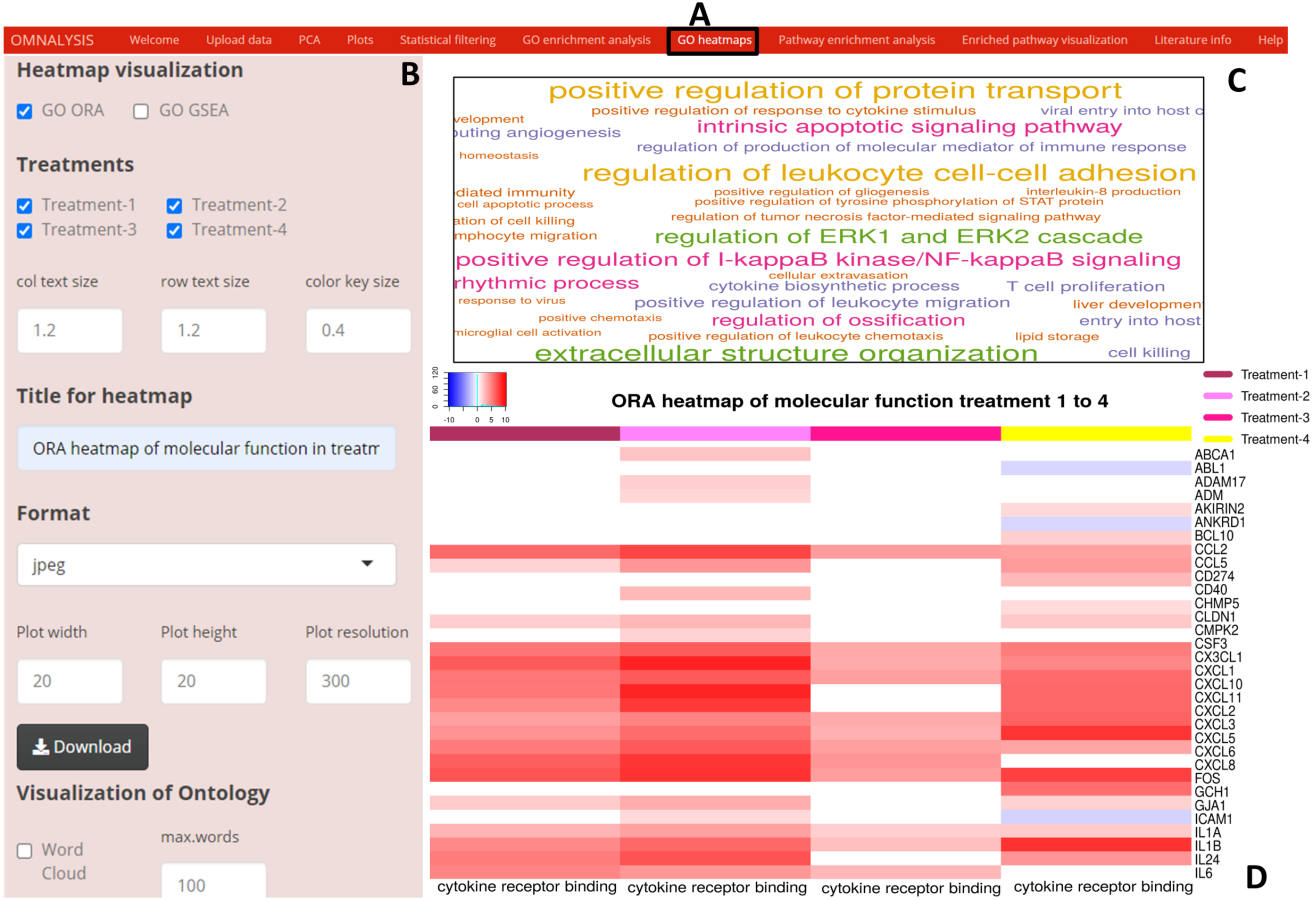
Enriched GO terms visualization using OMnalysis. (A) The user interface for GO heatmaps visualization. (B) An interactive panel to generate heatmaps and word cloud. (C) A word cloud providing an overview of GO terms enriched in treatment. The larger is the font size indicated more genes mapped to that GO term. (D) GO heatmap comparing gene expression among the treatment in a given GO term.

In the *GO heatmaps* UI tab (Fig. 5A) we included an interactive panel (Fig. 5B), which was populated with checkboxes for selection of heatmap visualization method, treatments checkboxes, numerical inputs boxes to adjust the font and color key size, word cloud checkbox to plot a word cloud, etc. The wordcloud package version 2.6 (Fellows, 2018) was employed to visualize a word cloud (Fig. 5C), while **heatmap.2** function of gplots version 3.1.1 (Warnes et al., 2016) and **rainbow** function of the R base version 4.0.3 (RStudioTeam, 2015) were used to generate an example heatmap (Fig. 5D).

### Pathway analysis

To get the mechanistic insight from the list of DEGs or DEPs, four different pathway analysis methods were explored (Fig. S2). To enrich the DEGs or DEPs against KEGG pathway database (Kanehisa & Goto, 2000), two functions namely **enrichKEGG** and **gseKEGG** from cluster profiler’s version 3.18.1 (Yu et al., 2012) were used.

The network topology analysis (NTA) (Alexeyenko et al., 2012), was used against reference databases such as biocarta (Rouillard et al., 2016), panther (Thomas et al., 2003), NCI- nature pathway interaction database (Anthony et al., 2011), and pharmGKB (Klein & Altman, 2004). For NTA, we used three function in R base version 4.0.3 namely **merge**, **cbind** and **gsub** (RStudioTeam, 2015) to arrange the data matrix of DEGs or DEPs. Then, we used the **graphite pathway** function of graphite package version 1.36.0 (GRAPH Interaction from pathway Topological Environment) (Sales et al., 2012) to prepare the reference pathway database. The graphite’s **runSPIA** function was used to perform network topology analysis using the four reference databases mentioned above.

An **enrichpathway** function of ReactomePA version 1.34.0 (Yu & He, 2016) was used to perform pathway analysis using the Reactome pathway database version 1.74.0 (Croft et al., 2011).

A **$new** function in STRINGdb version 2.2.2 (Szklarczyk et al., 2019) was used to assign the species, score threshold, and input directory. We used the **stringdb$map** function of the STRINGdb package to map the DEGs or DEPs against several databases (GO annotation, KEGG pathways, PubMed publications, Pfam domains, InterPro domains, UniProt Keywords SMART domains).

### Enriched pathway visualization

**Figure 6:**
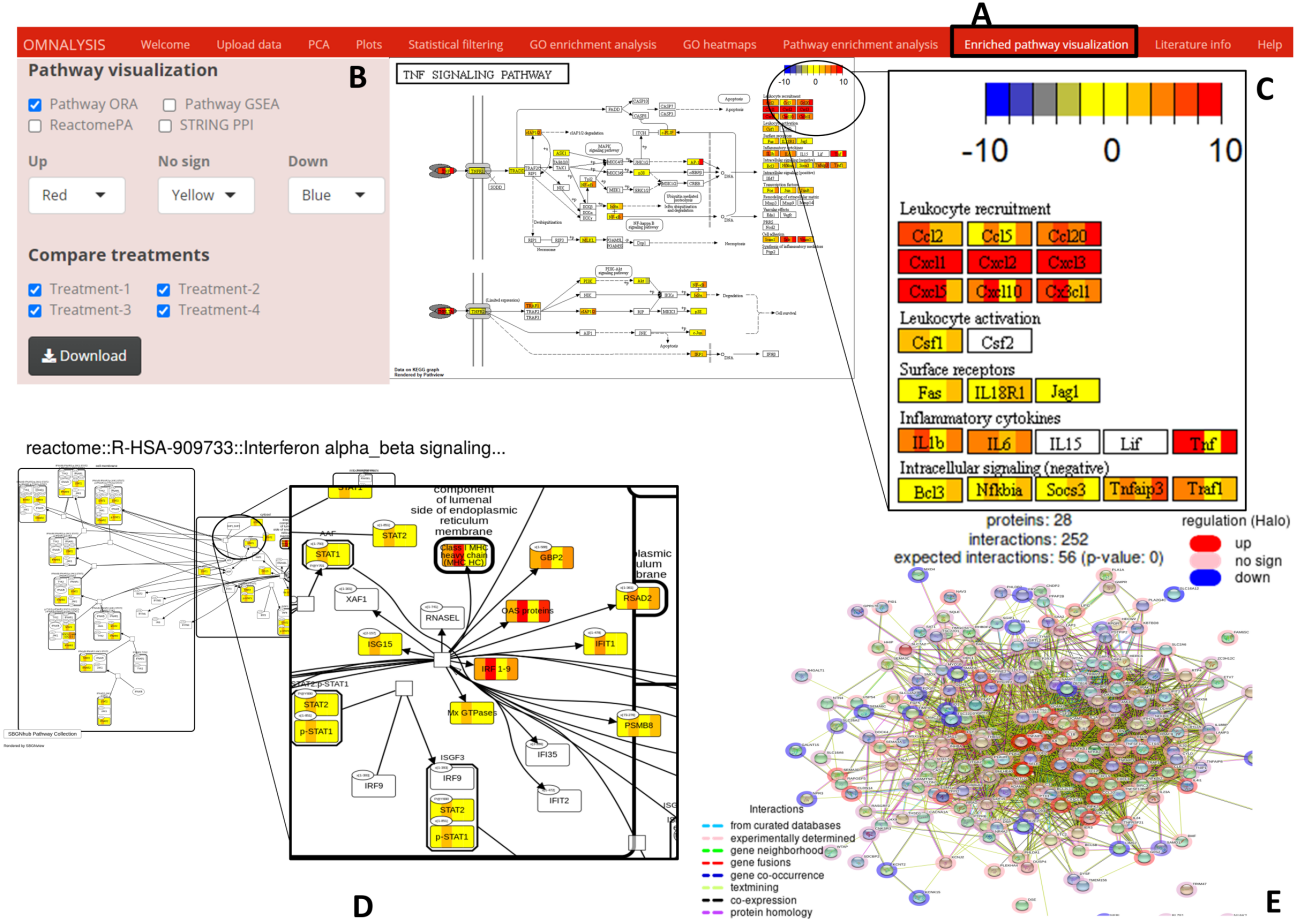
Enriched pathway visualization. (A) The user interface for pathway visualization. (B) An interactive panel to provide input for generating enriched pathway. Two types of KEGG pathways, one Reactome pathway, and a string PPI can be selected. In the interactive panel, various treatments can be selected for comparison and the generated pathways can be downloaded with a download button. (C) An example of a KEGG pathway generated. The pathway is showing the level of expression of each gene (red–up regulation, yellow- no significant expression, blue-down regulation) identified in all four treatments. Please note that the gene box is divided into the number of treatments automatically. (D) The expression value of the mapped genes to the Reactome pathway in all treatments. Each gene box is divided into 4 parts (each part showing the level of gene expression in color codes). (E) The protein–protein interaction network (STRING PPI). The level of expression is depicted with a colored halo.

*Enriched pathway visualization* tab of UI (Fig. 6A) was designed with an interactive panel (Fig. 6B) that contains pathway visualization checkboxes to select a type of enrichment method, a dropdown menu to select color code on the pathway, treatment checkboxes to compare the treatments. Pathview package version 1.30.1 (Luo & Brouwer, 2013) was used for the visualization of the pathways based on ORA or GSEA. An example of pathway is presented in Fig. 6C. SBGNview package (overlay omics data onto sbgn pathway diagrams) version 1.4.1 (Dong et al., 2021) was used to visualize the enriched pathway from ReactomePA (example is depicted in Fig. 6D). The **plot_network** and **post_payload** functions of STRINGdb package version 2.2.2 (Szklarczyk et al., 2019) were used to visualize the network of protein-protein interaction network (example is presented in Fig. 6E).

### Literature information

A europepmc package version 0.4 was used to fetch abstracts, publication details, and bibliography metadata in the *Literature info* UI tab of the OMnalysis web application.

### User manual

To help researchers, a user manual is drafted that provides step-by-step guidelines (Data S1).

## Results

### Workflow for transcriptomics and proteomics data analysis

We performed ID conversion on transcriptomics and proteomics data taken as example matrices using Ensembl ID and uniport ID, respectively. OMnalysis is built to assign the most updated IDs. To the transcriptomic data matrix, the OMnalysis assigned 11,357 updated Ensembl IDs from a total of 11,398 uploaded Ensembl ID (Table S7). Next, to those updated IDs, OMnalysis was successful in assigning 10,951 human gene names, 10,932 HGNC symbols, 11,354 gene descriptions, and 7,463 UniProtKB/Swiss-prot ID (Table S7). In the case of proteomic data, 731 UniProt IDs were submitted to OMnalysis. Please note that, these 731 IDs include several repeated UniProt IDs from 4 treatments. From this list, 281 UniProt IDs were mapped to Ensembl gene ID, 277 to the gene name, 0 to HGNC symbol, 273 to gene description and 273 to UniProtKB/Swiss-Prot ID (Table S8).

### Visualization, plotting and statistical filtering

When looking for the visualization, OMnalysis enables a user to generate separate plots as shown in Figs. 2–4 for each treatment. It generates PCA plots to identify the relationship and variability among the genes or proteins in the treatments. It produces scatter and volcano plots to visualize the significantly up and down regulated gene or proteins in each treatment. It makes Venn diagram to observe the intersection among genes or proteins and histogram to identify level of expression (logFC) of DEGs or DEPs in each treatment.

Further, OMnalysis provides the user functionality to filter out the genes or proteins which are not significantly expressed or identified. The software provides an option to set the cutoff values for logFC, logCPM, and *P* value for transcriptomic data (Fig. 4A), whereas logFC and *P* value for proteomic data. logFC ± 1.2, Log CPM >3, and *P* value <0.001 were set to select the DEGs from the transcriptomic data matrix. Whereas in proteomics logFC ±1.2 and *P* value <0.01 (FDR-adjusted *P*-value) were set to select the DEPs. The resultant list of the differentially expressed candidates was automatically transferred to the next level *i.e.*, the functional and pathway analysis.

### Functional and pathway analysis of example data

Gene ontology (GO) analysis were performed using ORA (at 0.05 *q*-value cutoff) and GSEA (0.5 *P* value cutoff) on the differentially expressed candidates selected above (Fig. S1). Using ORA (Table S9) and GSEA (Table S10), OMnalysis enriched the DEGs according to their biological process, molecular function, and cellular component. To minimize the chances of identification of false-positive GO IDs in ORA and GSEA, OMnalysis provides multiple hypothesis testing correction methods (Holm, Hochberg, Hommel, Bonferroni, Benjamini and Hochberg, BY and FDR) that enable adjustment of *P* value (Fig. S1). In OMnalysis, the user can select any single GO ID that is present in all the treatments and compare the expression of the gene with the heat maps. An example of the heatmap is presented in Fig. S3. In the same way, OMnalysis processed the set of the differentially expressed proteins (Fig. 4A and 4B) using ORA (0.05 *q*-value cutoff; Table S11) and GSEA (0.5 *P* value cutoff; Table S12) and enriched them according to the biological process, molecular function, and cellular component. OMnalysis can generate the same type of heatmaps as shown in Fig. S3 for proteomics.

For pathway analysis, OMnalysis used the list of differentially expressed candidates (DEGs and DEPs) and enriched them using ORA (Table S13) and GSEA (Table S14) against KEGG pathways (Fig. S2). For pathway enrichment analysis one of the multiple hypothesis testing correction methods can also be selected (Fig. S2). The network topology analysis (NTA) was also performed on OMnalysis to enrich the list of DEGs against various databases such as biocarta, panther, NCI, PharmGKB (Table S15). OMnalysis was also able to compare the list of differentially expressed candidates to the pathways available in Reactome (Table S16).

OMnalysis was extended to include the STRINGdb, which covers several databases (InterPro, SMART, PFAM domains, Reactome pathways, PubMed publications, UniProt Keywords, GO terms, and KEGG) to enrich the DEGs (Table S17).

One of the main features of OMnalysis is the visualization of the level of expression (using different color codes) of the given gene or protein in different treatments. As shown in Fig. S4 , the OMnalysis enriched DEGs from all four treatments to TNF signaling pathway (using ORA against KEGG pathways) and assigned color codes based on their expression values (logFC) in the gene box. This kind of representation gives a holistic view of the ongoing molecular activities simultaneously in all treatments in a given pathway. In the same way, OMnalysis used DEGs and visualized their level of elicitation on the enriched Reactome pathway (Fig. S5). To extend the functionality, OMnalysis visualizes the level of elicitation in protein-protein interaction network derived from STRINGdb in the form of halo color codes (Fig. S6).

In the case of the proteomics study, OMnalysis processed the list of DEPs using ORA (Table S18) and GSEA (Table S19) against the KEGG database. Whereas, the unavailability of supporting databases for bovine proteome hindered the pathway enrichment analysis using NTA and Reactome. While choosing STRINGdb, OMnalysis enriched the DEPs to InterPro, SMART, PFAM domains, Reactome pathways, PubMed publications, UniProt Keywords, GO terms, and KEGG (Table S20). For proteomics, OMnalysis can visualize the generated pathway in the same way as in Figs. S4–S6.

### OMnalysis output formats

OMnalysis was developed considering the maximum flexibility and customization of the resulting output. Users were provided with the option to download the converted accession IDs, results from GO enrichment analysis and pathway enrichment analysis in CSV file format. All the diagrams produced in *PCA*, *Plots*, *Statistical filtering*, and *GO heatmaps* sections can be downloaded in TIFF, PNG, JPEG, and PDF format. In the case of the enriched pathway section, the visualization output can be downloaded in PNG image format. Furthermore, users have the option to adjust the dimension and resolution of the images generated in each tab of OMnalysis.

## Discussion

Taking the advantage of Shiny, Bioconductor, flexdashboard, and markdown we were able to integrate and develop a user-friendly web tool OMnalysis. Using this tool, researchers can walk through various levels of exploration of quantitative data, which includes publication-ready plots, functional interpretation, pathway analysis, and scientific literature. Also, by leveraging the benefits of OMnalysis, the user will be able to analyze four differential expression data simultaneously, derived from quantitative transcriptomics or proteomics experiments. Till to date, few tools (iDEP, DEBrowser, IRIS-EDA, etc.) are developed, which accept count data to analyze differential expression, however, this approach is complicated for the biologist in terms of selecting the normalization methods. The normalization methods that available are CPM (count per million), TPM (transcripts per kilobase million), FPKM/RPKM (fragment/reads per kilobase of transcript per million mapped reads), DESeq2 (median of rations) (Love, Huber & Anders, 2014), and EdgeR (trimmed mean of M values) (Robinson, McCarthy & Smyth, 2010). Hence, biologists often use data matrices that contain a list of genes or proteins with expression values and statistical components for downstream differential expression analysis. Although the later option inherits some limitations *e.g.*, inability to perform differential expression analysis using count or spectral data and lack of metadata table, it benefits the larger research community by minimizing the time to obtain the result and to understand the normalization methods. Some tools like iDEP (Dijk et al., 2018), ShinyNGS (Manning, 2016) and DEBrowser (Kucukural et al., 2019) require additional metadata table to provide the information related to samples and study design. To this background, the OMnalysis is built to provide the researcher with a user-friendly web application, with no metadata dependency, and with streamlined analysis tabs. It covers peer-reviewed, curated, and updated databases, and it enables advanced visualization in form of plots, mapping of the expression data (logFC) on pathways and networks using pseudo colors. A single enriched pathway decorated with the colored map of expression value, provides the user with a holistic view of biological activities in different treatments.

We compared the OMnalysis with existing freeware used for DEGs analysis and exploration (*e.g.*, iDEP, IRIS-EDA, START app, DEBrowser, etc.), in terms of input data requirements, types of visualization, ease of use, and database used for GO and Pathway enrichment analysis. The details of this comparison are provided in Table 2. In contrast to OMnalysis, IRIS-EDA and START app does not support gene ID conversion. The iDEP web application requires a manual update of the database to support gene ID conversion. DEBrowser although performs batch effect correction and DEGs analysis using count data, it requires an R environment to generate a web interface, which could be a bottleneck in analysis for most biologists as they hold minimum programming knowledge. In contrast, OMnalysis is an online application and doesn’t depend on the R environment.

**Table 2:** Comparison of a bioinformatics platform for downstream analysis.

	iDEP	IRIS-EDA	START App	ShinyNGS	DEBrowser	OMnalysis
Input data	Count data, meta-data	Count data	Count or expression data	Count or expression data	Count data	Expression data (list of DEGs or Proteins)
PCA	√	√	√	√	√	√
Volcano/Scatter plot	√	√	√	X	√	√
GO analysis	√	X	X	√	√	√
Pathway analysis	√	X	X	X	√	√
Gene ID conversion	√	X	X	X	X	√
Pathway databases	KEGG, STRING API	X	X	KEGG	KEGG	KEGG, Reactome, NCI, Panther, biocarta, PharmGKB, STRING
Literature retrieval	X	X	X	X	X	√
Application type	Online	Online	Online	Require R for online	Require R for online	Online
Stand-alone R code	√	√	√	√	√	√
Pathway visualization and STRING network	√	X	X	X	√	KEGG, Reactome, PPI network

**Notes.**

X and √ indicates non-available and available function in the tool, respectively.

When it comes to the representation of data in the form of scatter and volcano plots to demonstrate the level of expression against the level of significance (*P* value) or the number of transcripts (logCPM), the OMnalysis provides an option with customizable resolution and dimensions of the images, and enables various image format to download (png, jpeg, tiff, and pdf). The iDEP generates scatter and volcano plots only in esp format, whereas the IRIS-EDA and START applications produce plots only in png formats. For statistical filtering of non-significant genes, the IRIS-EDA uses adjusted *P* value and fold change, whereas in OMnalysis can filter out the non-significant genes based on fold change, *P* values, or adjusted *P* value and log counts per million in case of transcriptomic data.

In the IRIS-EDA tool, the enrichment analysis and functional interpretation are extended by providing the weblinks of third-party web servers (DAVID, UCSC Genome Browser, etc.). Whereas, in OMnalysis the functional interpretation and enrichment analysis is integrated and supported by ORA and its extension GSEA. ORA uses the hypergeometric test (Falcon & Gentleman, 2008) and GSEA uses the Kolmogorov–Smirnov statistics (Smirnov, 1948) to perform GO enrichment analysis. ORA and GSEA perform multiple hypothesis testing using the gene set against the GO dataset, however in each run it may add some false-positive results. To control false positives, OMnalysis supports adjustment of the *P* value using multiple hypothesis correction methods (*e.g.*, Holm, Hochberg, Hommel, Bonferroni, Benjamini and Hochberg, BY and FDR) and a *P* value (*P* value cutoff) to gain more reliable information. OMnalysis also provides options to segregate the DEGs or DEPs into various GO classes (biological processes, molecular functions, cellular component (Ashburner et al., 2000)), which is not available in the IRIS-EDA tool. Note that, when interpreting the result from GO and pathway enrichment analysis, a user must be cautious and try a combination of methods and databases to obtain the comprehensive result. Thus, OMnalysis integrated approach provides the user to perform the enrichment analysis and functional prediction in one web application.

Heatmaps are one of the best representation tools to compare the level of expression among various treatments. Users often use third-party heatmapper (Babicki et al., 2016) web application, which requires an input (in manually arranged tabular form) populated with columns such as unique gene or protein IDs, GO IDs, and expression values for each treatment. To this end, we integrated the Heatmap function in the OMnalysis that automatically arrange the table and generates the heatmaps. It also identifies duplicate gene or protein names and filter out those redundancies. Furthermore, in comparison to IRIS-EDA and START app, OMnalysis provides customizable (key size and font size) and downloadable publication-ready heatmaps.

Finally, using the R Shiny platform and Bioconductor packages, we were able to integrate the several functionalities into OMnalysis. The streamlined functionalities include uploading of expression data, PCA to identify correlation and variability among treatments, plots to visualize differential expressions, statistical filtering to segregate the candidate according to the statistical significance, GO enrichment analysis, heatmaps to compare expression among treatments, pathway enrichment analysis, and pathway visualization capabilities. All together the OMnalysis provides the user with a comprehensive explanation of the transcriptomics and proteomics data. To our knowledge, no integrated web tool provides visualization of pathways based on KEGG and Reactome, and visualization of PPI network using STRING in one place. OMnalysis with higher flexibility, easy-to-use interface, multiple visualizations, and extensive coverages of curated databases outperforms many of the currently available web application available to explore and analyze the quantitative transcriptomics and proteomics data.

## Conclusion

OMnalysis has integrated an array of scattered packages and curated databases to provide a user-friendly data analysis tool. The overall functionality was tested on the four real datasets of transcriptomics and proteomics. Using these datasets, we were able to perform series of downstream analysis, starting from PCA and visualization of differentially expressed candidates in single or multiple treatments in the form of scatter or volcano plots, Venn diagram and histogram. Further, this tool was able to segregate gene sets based on any of the three gene ontology classes (biological processes, molecular functions, cellular component) with seven possible multiple hypothesis correction methods and two types of enrichment analysis (ORA and GSEA). This tool provided different view on transcriptomics and proteomics data using three enrichment methods (ORA, GSEA and ReactomePA) and network topology analysis using four different databases (PANTHER, biocarta, NCI and PharmGKB). Additionally, STRING gave overall picture of enrichment and interaction among molecules. Comparing with the other tools, OMnalysis provides more customizable and functional options. We envisage developing an advanced version of OMnalysis, which will include more animal species, omics types, additional pathway networks (*e.g.*, Wiki pathways, Pathbank, etc.), and characterization of functional units of discovered biomarkers (genes, proteins, and metabolites). Currently, we have added an option to download the set of codes, so that bioinformaticians can extend the functionality of the OMnalysis tool. With the existing capabilities, we are confident that OMnalysis will be a useful web application for researchers, with no or less bioinformatics experience, who want to analyze quantitative transcriptomic and proteomic data into actionable biological insight.

## Supplemental Information

10.7717/peerj.12415/supp-1 Supplemental Information 1 Transcriptomics data of *Borrelia burgdorferi*

10.7717/peerj.12415/supp-2 Supplemental Information 2 Transcriptomics data of *Neisseria meningitidis*

10.7717/peerj.12415/supp-3 Supplemental Information 3 Transcriptomics data of *Streptococcus pneumoniae*

10.7717/peerj.12415/supp-4 Supplemental Information 4 Transcriptomics data of West Nile Virus

10.7717/peerj.12415/supp-5 Supplemental Information 5 Compiled transcriptomics data

10.7717/peerj.12415/supp-6 Supplemental Information 6 Proteomics data

10.7717/peerj.12415/supp-7 Supplemental Information 7 Transcriptomics ID conversion table

10.7717/peerj.12415/supp-8 Supplemental Information 8 Proteomics ID conversion table

10.7717/peerj.12415/supp-9 Supplemental Information 9 Transcriptomics ORA GO enriched table for GO classes

10.7717/peerj.12415/supp-10 Supplemental Information 10 Transcriptomics GSEA GO enriched table for GO classes

10.7717/peerj.12415/supp-11 Supplemental Information 11 Proteomics ORA GO enriched table for GO classes

10.7717/peerj.12415/supp-12 Supplemental Information 12 Proteomics GSEA GO enriched table for GO classes

10.7717/peerj.12415/supp-13 Supplemental Information 13 Transcriptomics ORA KEGG pathway enriched table

10.7717/peerj.12415/supp-14 Supplemental Information 14 Transcriptomics GSEA KEGG pathway enriched table

10.7717/peerj.12415/supp-15 Supplemental Information 15 Transcriptomics NTA table

10.7717/peerj.12415/supp-16 Supplemental Information 16 Transcriptomics Reactome pathway enriched table

10.7717/peerj.12415/supp-17 Supplemental Information 17 Transcriptomics STRING enriched table

10.7717/peerj.12415/supp-18 Supplemental Information 18 Proteomics ORA KEGG pathway enriched table

10.7717/peerj.12415/supp-19 Supplemental Information 19 Proteomics GSEA KEGG pathway enriched table

10.7717/peerj.12415/supp-20 Supplemental Information 20 Proteomics STRING enriched table

10.7717/peerj.12415/supp-21 Supplemental Information 21 User interface tab to perform GO enrichment analysis

10.7717/peerj.12415/supp-22 Supplemental Information 22 User interface tab to perform pathway enrichment analysis

10.7717/peerj.12415/supp-23 Supplemental Information 23 Example of GO heatmap generated from transcriptomics data

10.7717/peerj.12415/supp-24 Supplemental Information 24 Example of an enriched KEGG pathway The top right color key presents the range of logFC values. The OMnalysis has divided gene boxes into four sections, each presenting one treatment with pseudo color according to the level of expression.

10.7717/peerj.12415/supp-25 Supplemental Information 25 Example of expression profile on enriched Reactome pathway The top right color key presents the range of positive and negative logFC. The Gene box in the pathway is divided into four sections, each presenting one treatment with pseudo color according to the level of expression.

10.7717/peerj.12415/supp-26 Supplemental Information 26 Example of the protein-protein interaction network PPI network generated by OMnalysis. Each node presents a gene with the log fold change value in blue - downregulation, red - upregulation. Edges in the network provide evidence of the interactions between the genes or proteins. The color code of the edges is provided in the figure.

10.7717/peerj.12415/supp-27 Supplemental Information 27 A step-by-step guide to reproduce the OMnalysis workflow results

## Additional Information and Declarations

### Data Availability

The following information was supplied regarding data availability: The OMnalysis codes are written in R language and available at GitHub: https://github.com/Punit201016/OMnalysis. OMnalysis is also available at: http://lbmi.uvlf.sk/omnalysis.html. OMnalysis is hosted on a Shiny server at https://omnalysis.shinyapps.io/OMnalysis/. The minimum system requirements are: 4 gigabytes of RAM, i3 processor (or equivalent). It is compatible with all operating systems (Windows, Linux, Mac) and works on all browsers.
