# Supplementary figures and images for: Development of a bioinformatics platform for analysis of quantitative transcriptomics and proteomics data: the OMnalysis
Source: PeerJ. 2021 Nov 9;9:e12415. doi: 10.7717/peerj.12415 (PMC8588854; doi:10.7717/peerj.12415)

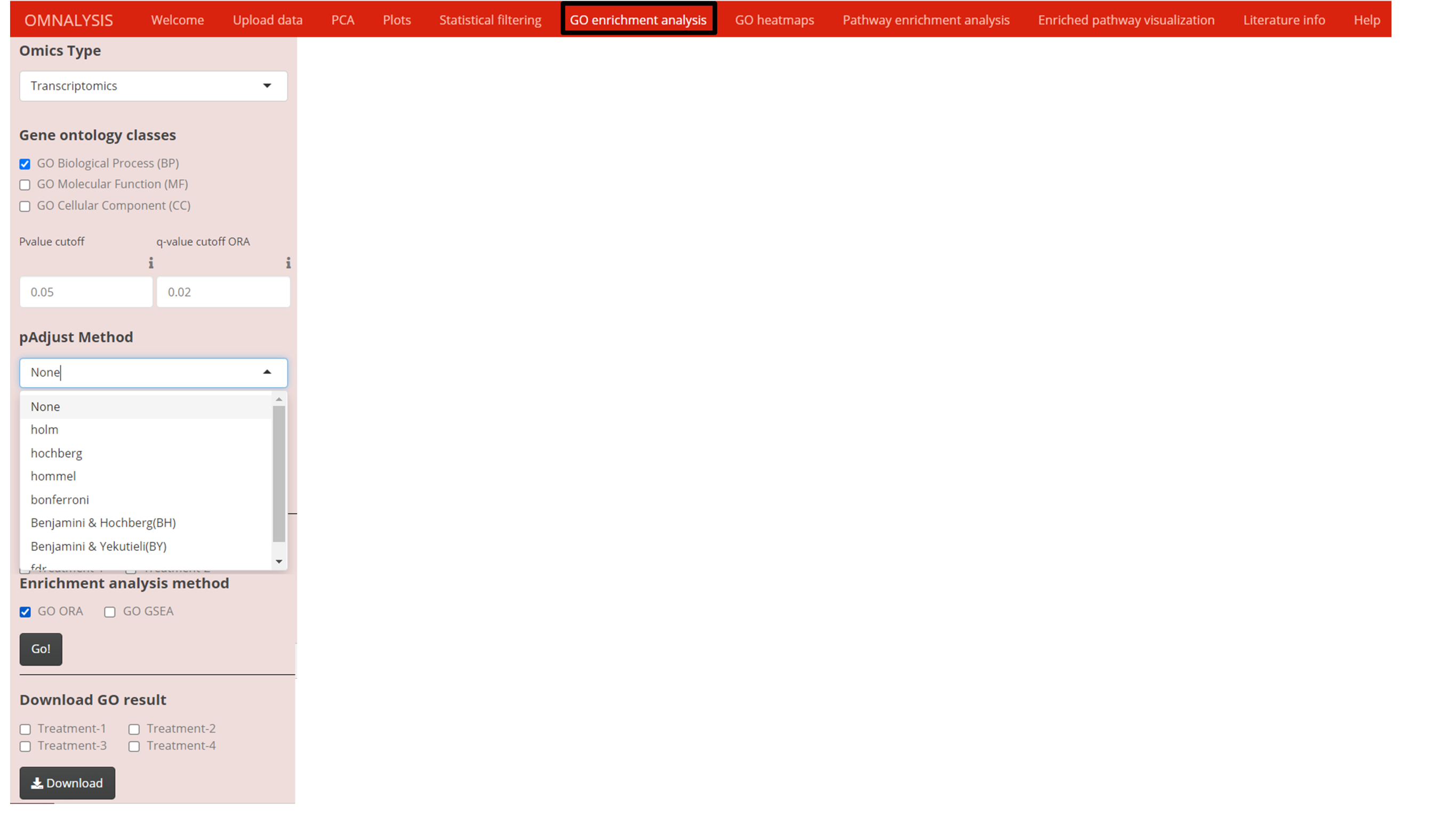

Supplement: Supplemental Information 21 [file peerj-09-12415-s021.png]

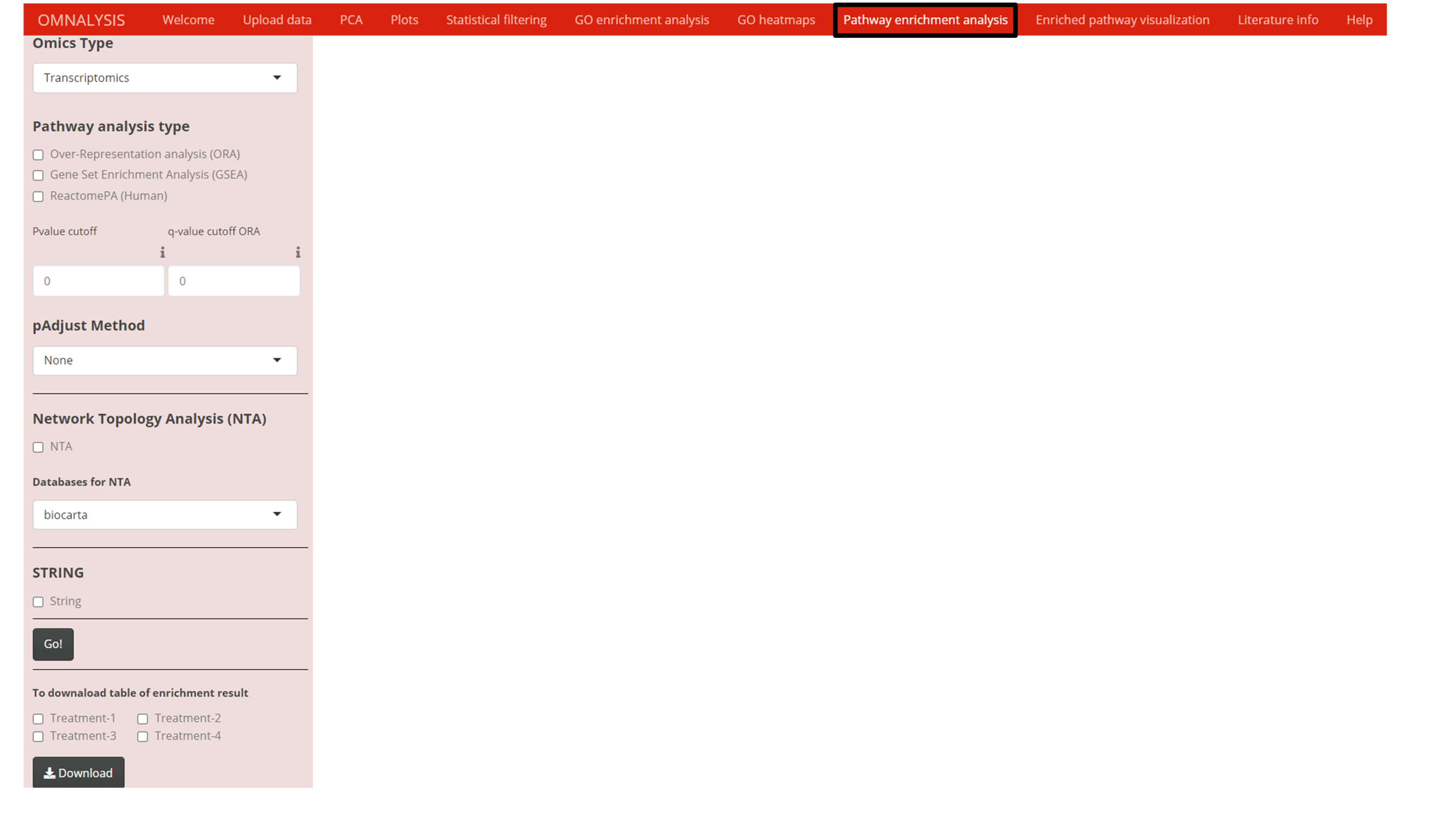

Supplement: Supplemental Information 22 [file peerj-09-12415-s022.png]

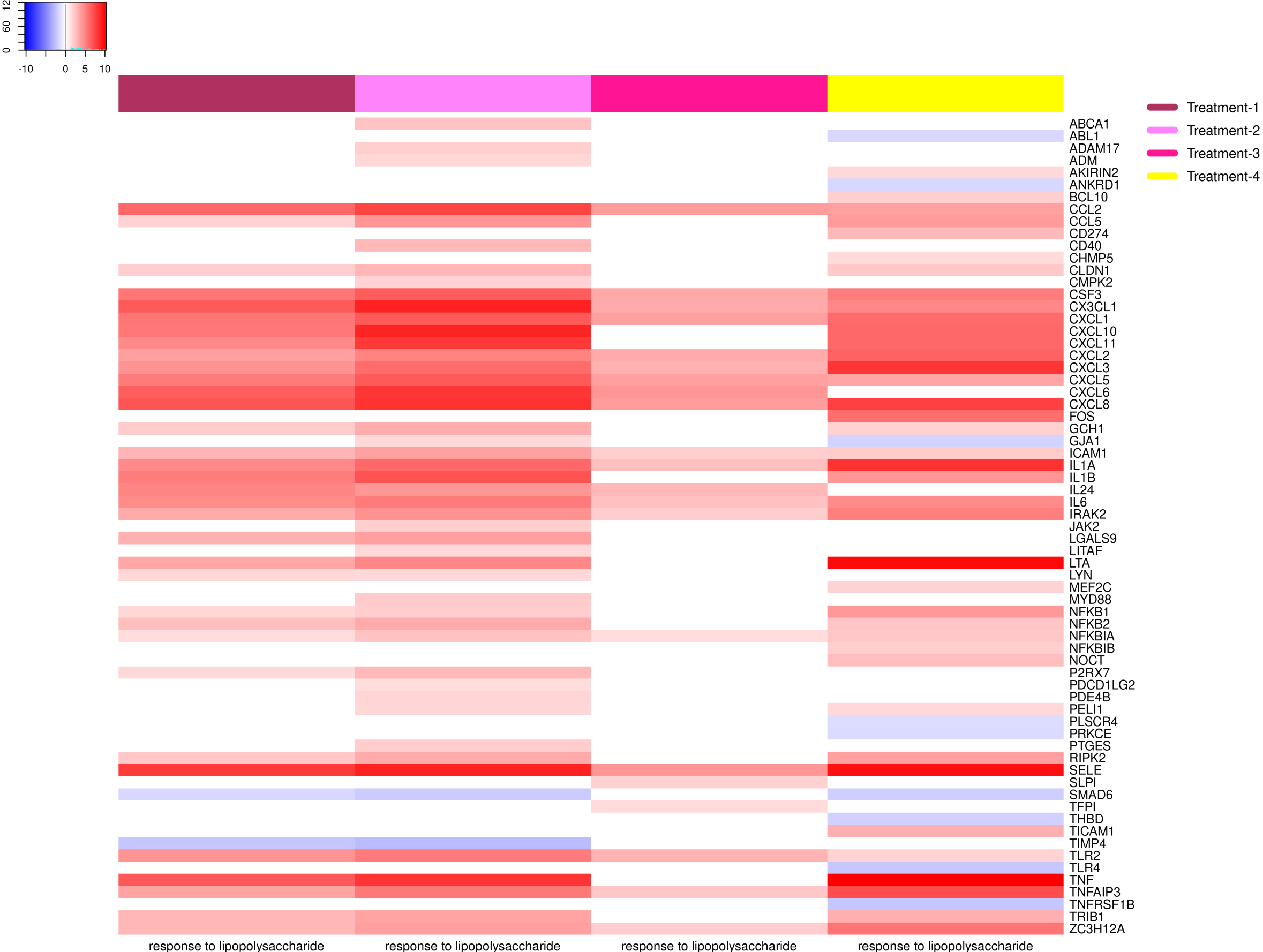

Supplement: Supplemental Information 23 [file peerj-09-12415-s023.png]

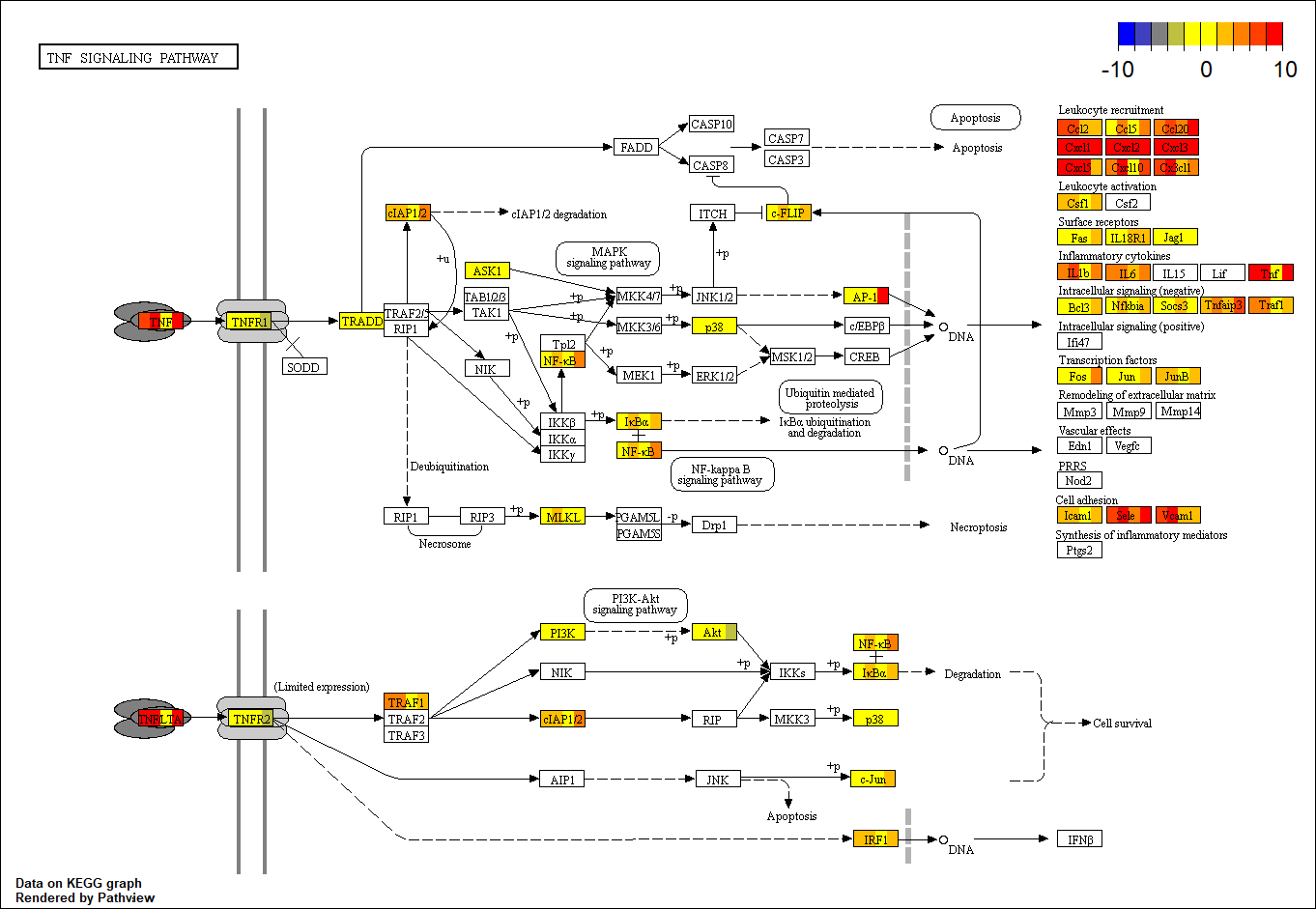

Supplement: Supplemental Information 24 — The top right color key presents the range of logFC values. The OMnalysis has divided gene boxes into four sections, each presenting one treatment with pseudo color according to the level of expression. [file peerj-09-12415-s024.png]

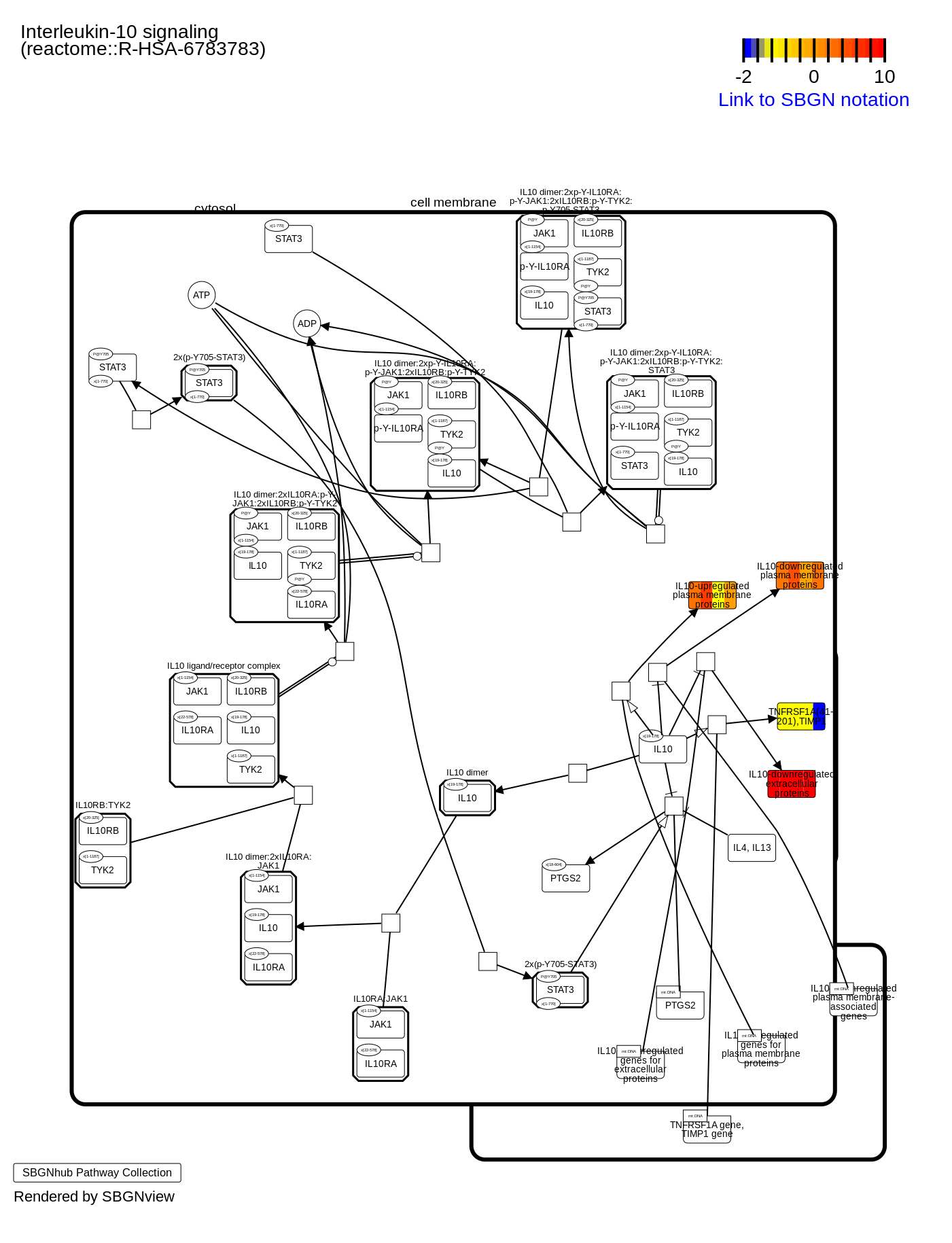

Supplement: Supplemental Information 25 — The top right color key presents the range of positive and negative logFC. The Gene box in the pathway is divided into four sections, each presenting one treatment with pseudo color according to the level of expression. [file peerj-09-12415-s025.png]

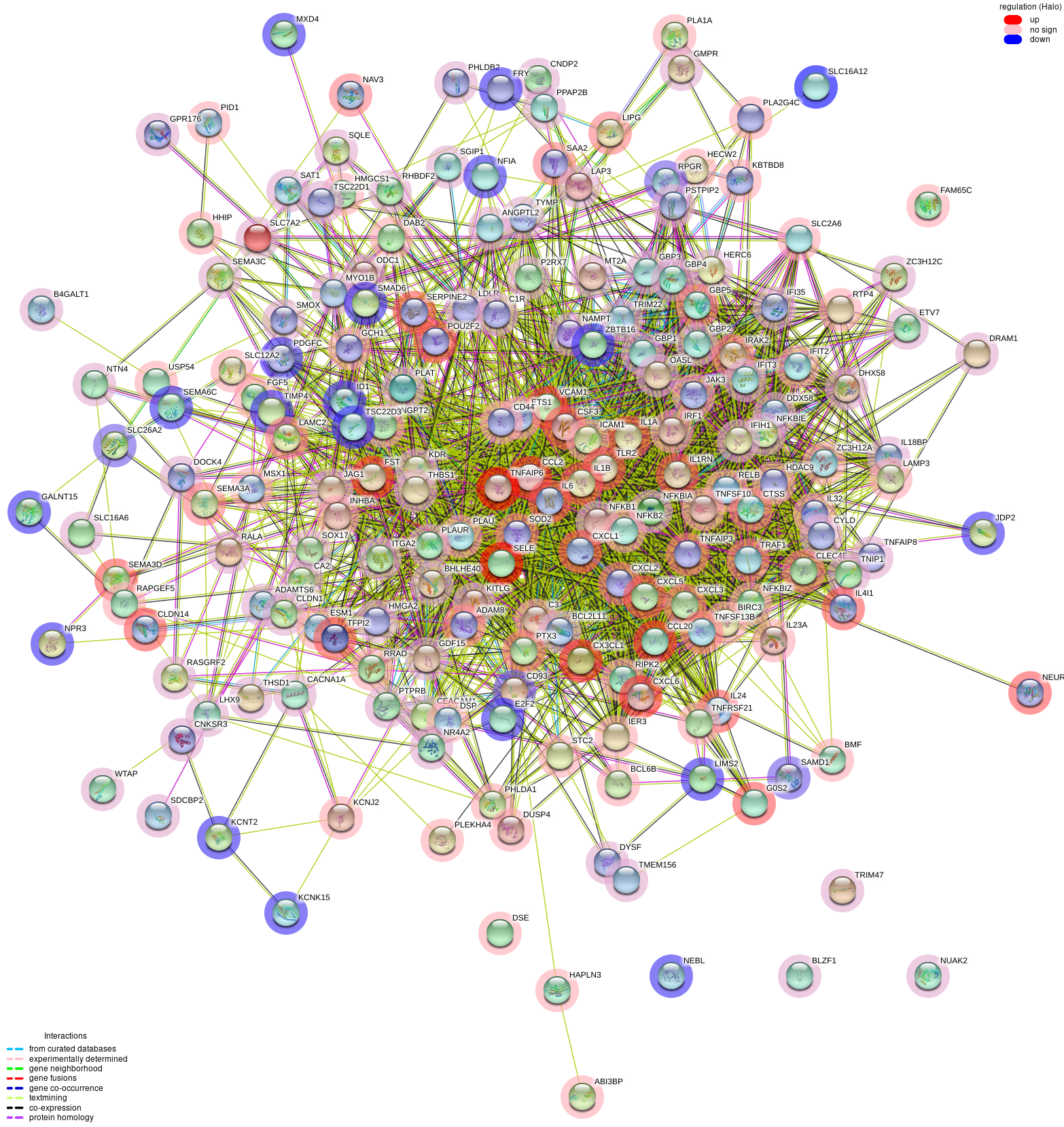

Supplement: Supplemental Information 26 — PPI network generated by OMnalysis. Each node presents a gene with the log fold change value in blue - downregulation, red - upregulation. Edges in the network provide evidence of the interactions between the genes or proteins. The color code of the edges is provided in the figure. [file peerj-09-12415-s026.png]
