# Supplementary material for: Development of a bioinformatics platform for analysis of quantitative transcriptomics and proteomics data: the OMnalysis
Source: PeerJ. 2021 Nov 9;9:e12415. doi: 10.7717/peerj.12415 (PMC8588854; doi:10.7717/peerj.12415)
Supplement: Supplemental Information 27 [file peerj-09-12415-s027.docx]

**Development of a bioinformatics platform for analysis of transcriptomics and quantitative proteomics data: OMnalysis**

User manual

Contents

[Introduction 3](#_Toc82092020)

[Instructions 4](#_Toc82092021)

[Data format 4](#_Toc82092022)

[Upload data 6](#_Toc82092023)

[PCA 8](#_Toc82092024)

[Plots 9](#_Toc82092025)

[Statistical filtering 10](#_Toc82092026)

[Gene ontology (GO) enrichment analysis 11](#_Toc82092027)

[GO heatmaps 12](#_Toc82092028)

[Pathway enrichment analysis 13](#_Toc82092029)

[Enriched pathway visualization 14](#_Toc82092030)

[Literature info 17](#_Toc82092031)

# Introduction

OMnalysis is developed using R shiny, flexdashboard and Bioconductor packages. This tool target researchers who are new to RNA-seq technology and proteomics study and often depends on commercial vendors or core facility to sequence and analyze their data. Although exploration of the identified list of genes and proteins is also tedious and challenging (*Fig.1*).

There are open-source R packages such as DESeq2 and edgeR that are extensively used to identify differentially expressed genes from the count data. OMnalysis uses the list of genes produced from one of the differential expression analysis software edgeR’s glmTreat function with count per million normalizations, significance value ( *P* value) and log fold change value assigned to each list of a gene in the treatment (*Fig. 2*). On the other hand, label-free relative quantitative proteomics data must contain the columns of UniProt ID, FDR-adjusted P-value and Fold Change in an excel file with each sheet of an experimental condition named Treatment1, Treatment2, Treatment3 and Treatment4 (*Fig. 3*).


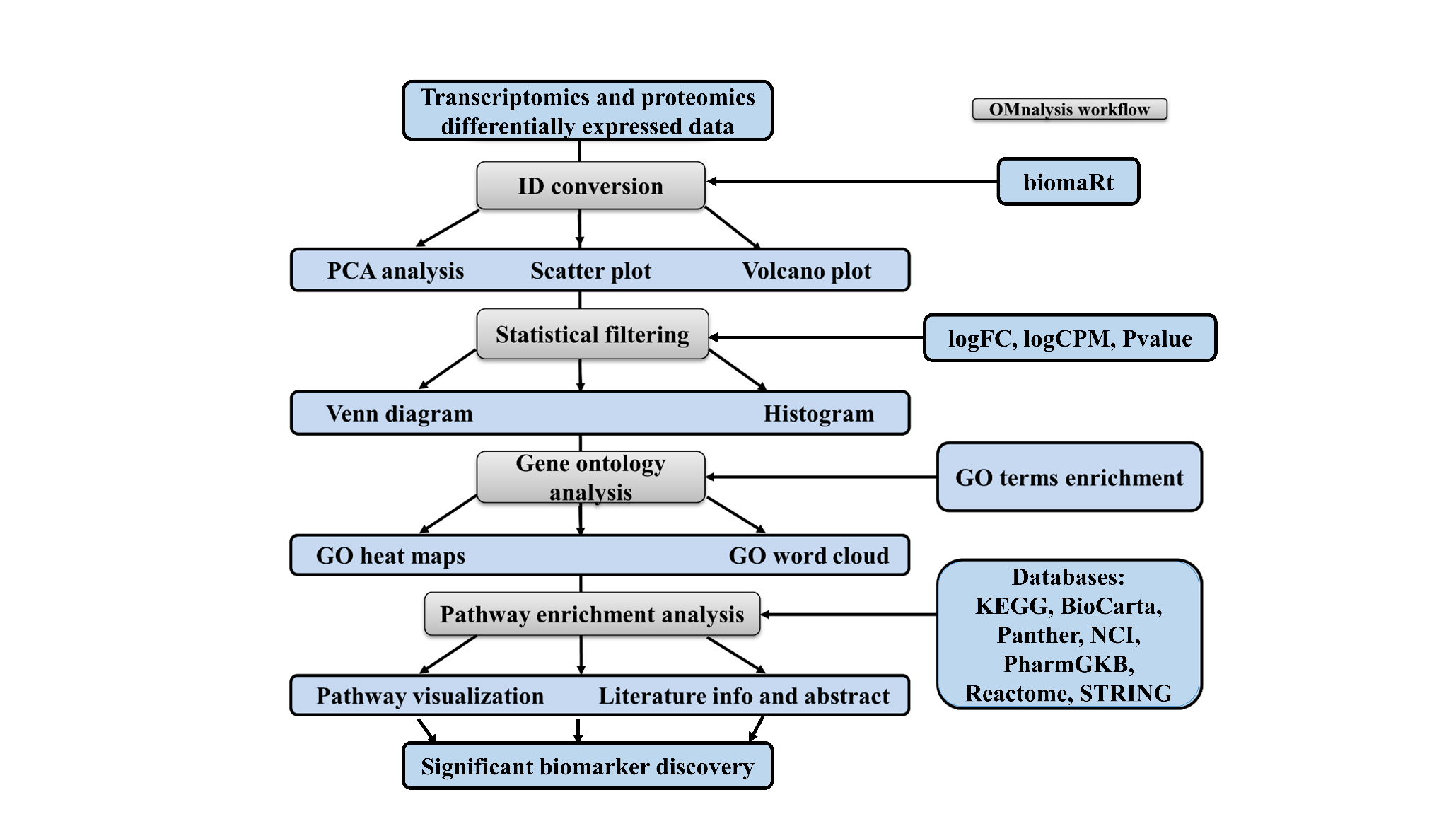


The app is hosted on shiny.io link <https://omnalysis.shinyapps.io/OMnalysis/>

# Instructions

1. You can run this app on your desktop after installing R base version4.0.3 and R studio version 1.4.1106.
2. Once the R environment is ready, install below packages required for OMnalysis.
3. Install R shiny supporting packages.
4. install.packages(c("flexdashboard", "dplyr", "shiny", "shinydashboard", "DT", "tidyverse", "shinythemes", "tidyr", "gplots", "tibble", "gridExtra", "RColorBrewer", "slickR", "devtools", "ggbiplot", "factoextra", "ggplot2", "data.table", "VennDiagram", "fields", "wordcloud", "SBGNview", "europepmc", "shinyjs", "futile.logger", "rio", "plyr"))
5. Install Bioconductor packages version 3.12 using Install.packages("BiocManager")
6. install.packages(c("AnnotationDbi", "Biobase", "BiocFileCache", "BiocGenerics", "BiocParallel", "BiocVersion", "biomaRt", "Biostrings", "clusterProfiler", "DO.db", "DOSE", "EnhancedVolcano", "enrichplot", "fgsea", "GO.db", "GOSemSim", "graph", "graphite", "IRanges", "KEGGgraph", "KEGGREST", "org.Bt.eg.db", "org.Gg.eg.db", "org.Hs.eg.db", "org.Ss.eg.db", "pathview", "qvalue", "reactome.db", "ReactomePA", "Rgraphviz", "S4Vectors", "XVector", "zlibbioc", "STRINGdb", "SPIA", "SBGNview"))

# Data format

**Steps for transcriptomics: -**

1. The differential data from the core facility or the commercial vendors will be in edgeR.tsv format.
2. Before going further make copies of edgeR.tsv files in one folder and rename them as per treatments.
3. Open edgeR.tsv file for each treatment in excel. Select and highlight columns belonging to ensemble ID (column A below), logCPM, log2Fold change and pvalue or padj as shown below


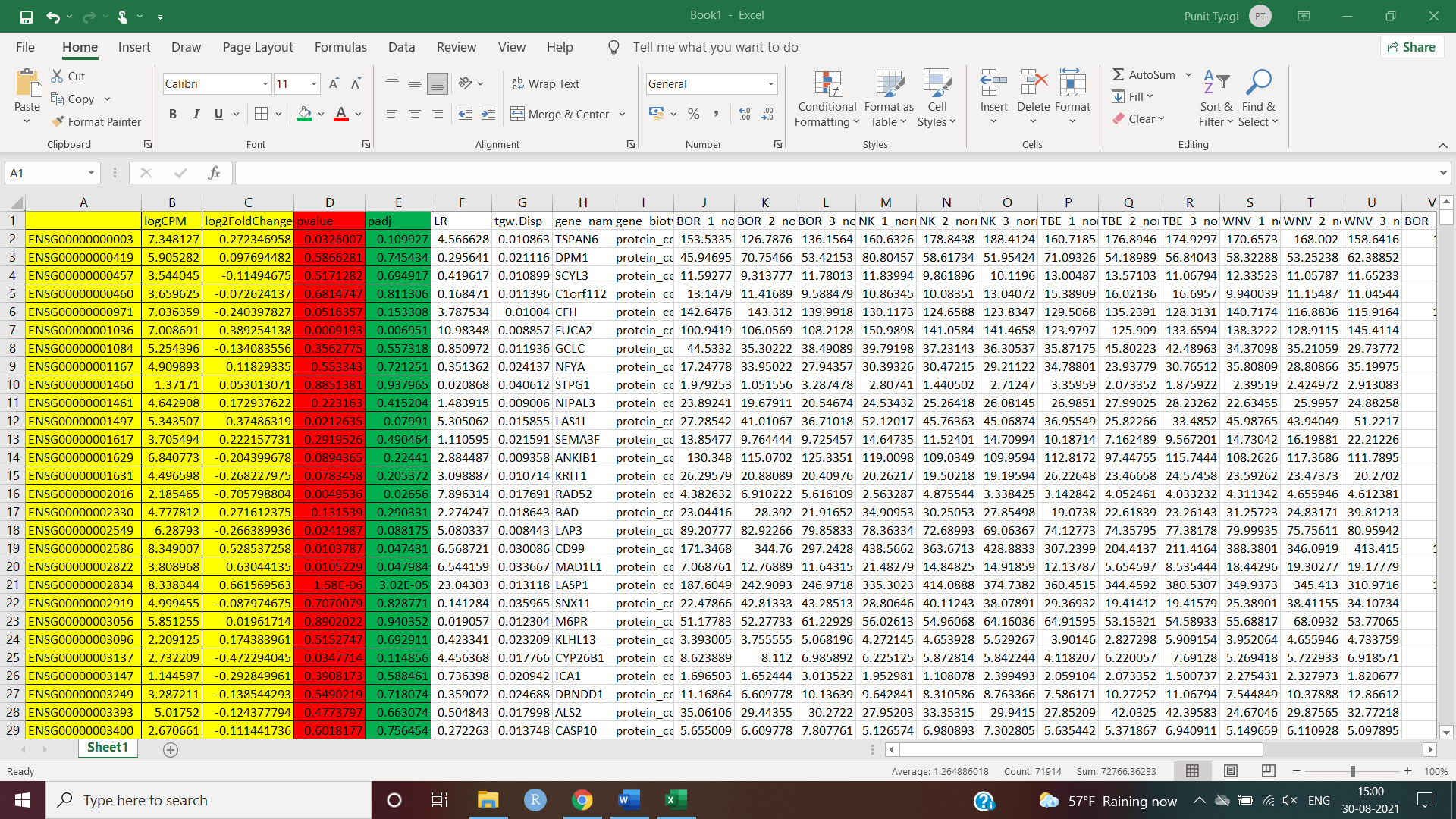


1. Delete all other columns.
2. sort column A in A-to-Z (sorting with expanded selection mode).


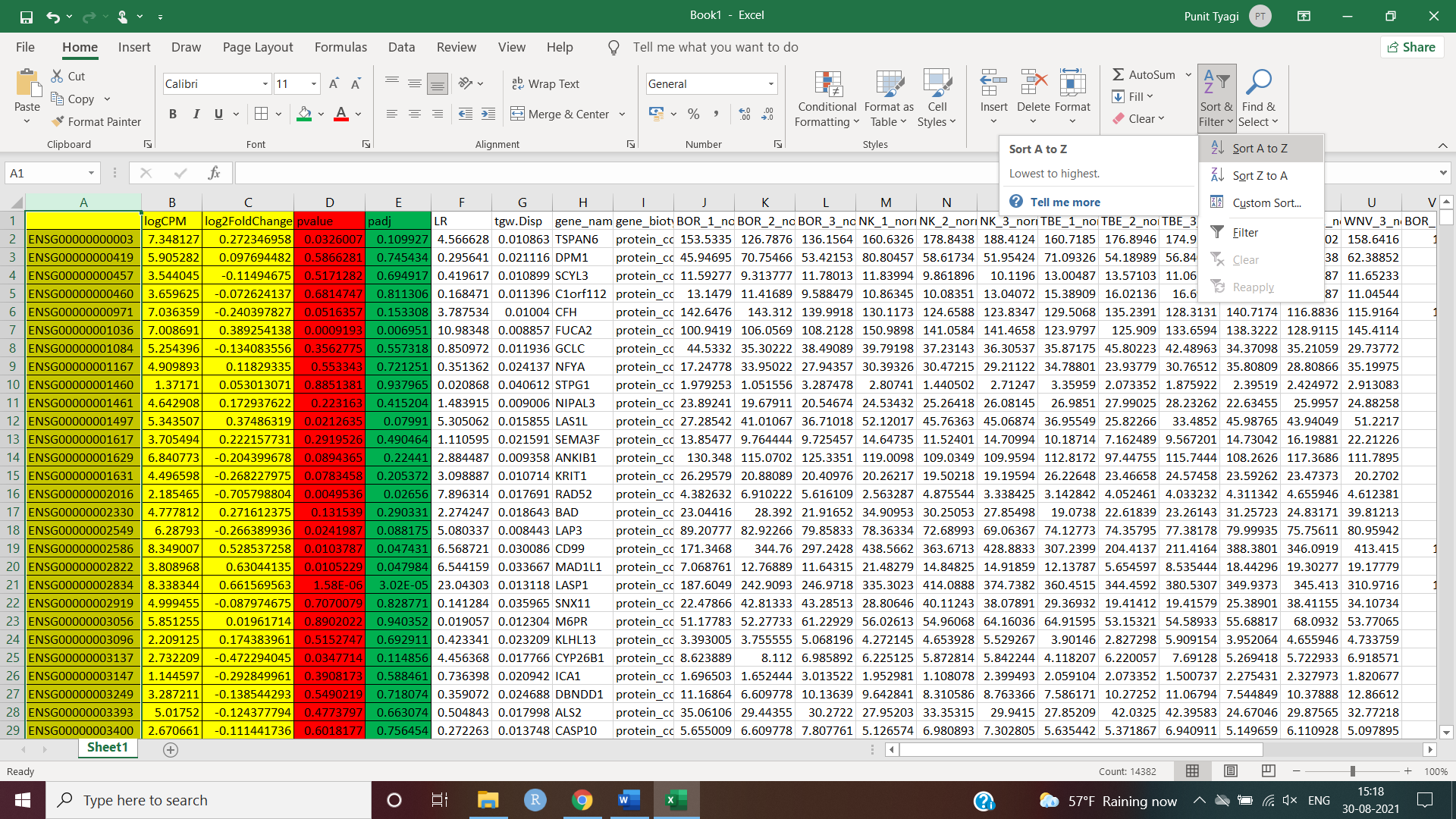


1. Repeat points 3 to 5 for each treatment edgeR.tsv file.
2. After sorting the information according to column A (yellow), check the first and last ensemble gene ID in all the treatments (it must be the same).
3. Open a template excel file ( ) and paste column A (yellow) of the first treatment and name the column header as ENSEMBLGENE.
4. Copy in order log2FoldChnage column C (yellow), logCPM column B and pvalue column D (red) or padj column E (green) from each treatment excel file.
5. Paste the copied columns next to the ENSEMBLGENE in the template excel file.
6. **The master file must look like the below screenshot.** Yellow color columns belong to treatment 1, green color columns belong to treatment 2, red color columns belong to treatment 3 and blue color columns belong to treatment 4.


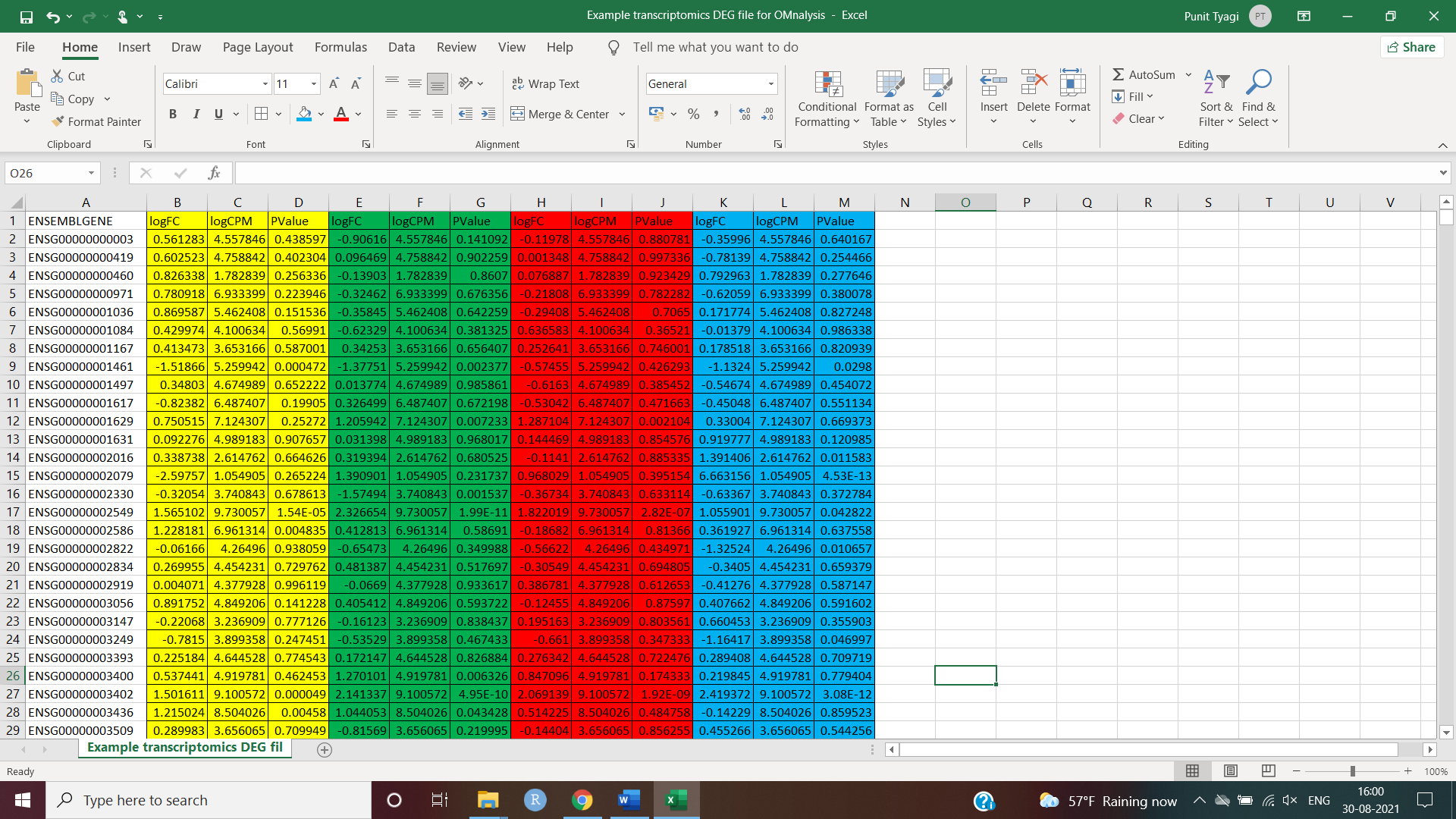


1. Column headers and the order of the columns (logFC, logCPM, Pvalue) must be the same as shown in the above screenshot.

**Steps for Proteomics: -**

1. The output from the differential proteomics analysis pipeline for each treatment is in a separate xlsx file.
2. Open each treatment file in excel.
3. Each treatment file must contain the column headers, UniProt ID, FDR-adjusted P-value and Fold Change in the same order presented in the below screenshot.


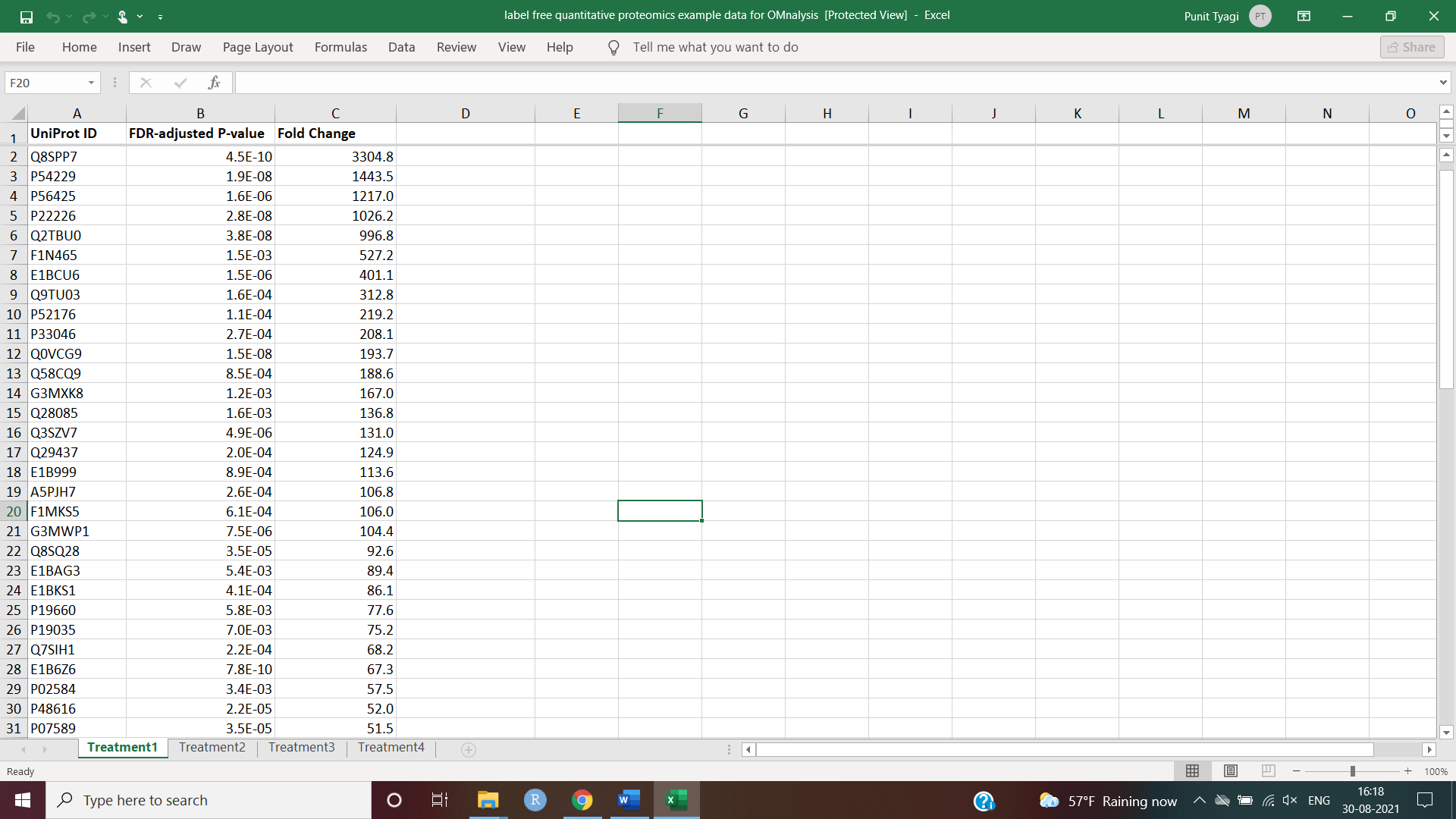


1. Open a new .xlsx file (master file) and name the first excel sheet as Treatment1 (Screenshot below)


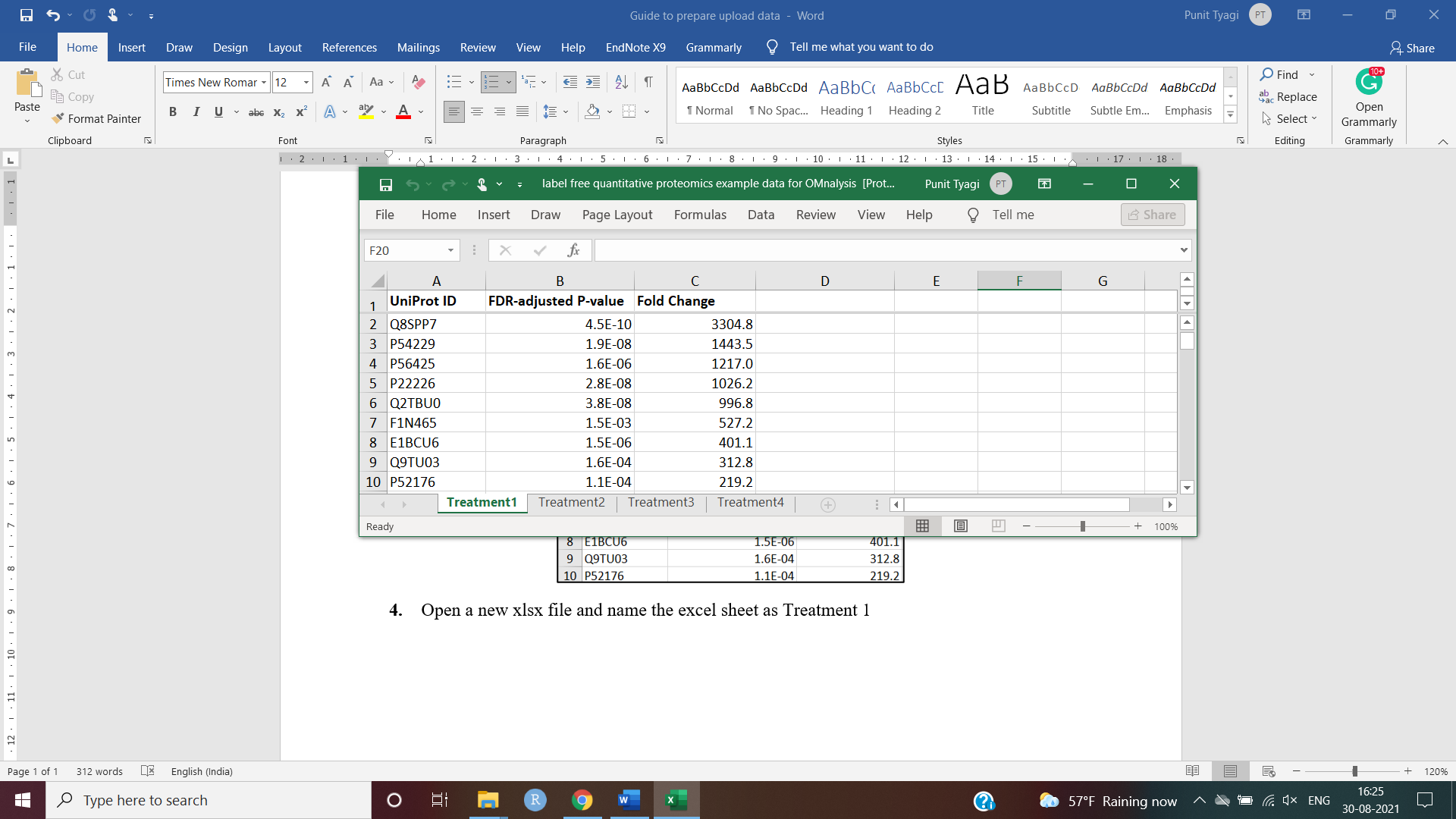


1. Paste the columns from each treatment into the master file with a new excel sheet and naming them as Treatment1 for the first treatment, Treatment2 for the second treatment, Treatment3 for the third treatment, Treatment4 for the fourth treatment.
2. Save the master file in .xlsx format.

# Upload data

1. The app features can be explored using the **Click to upload transcriptomics example data tab for transcriptomics analysis and Click to upload Proteomics example data for proteomics data analysis**.
2. The interactive panel below (left side), use the pre-loaded RNA-Seq data produced by human brain microvascular endothelial cells (hBMEC) induced with various pathogens: *Borrelia burgdorferi* (Treatment1, *Table S1*), *Neisseria meningitidis* (Treatment2, *Table S2*)*, Streptococcus pneumoniae* (Treatment3, *Table S3*), and West Nile Virus (Treatment4, *Table S4*). Tables are available in the supplemental information.
3. In the interactive panel below, use the pre-loaded label-free proteomics example data of milk whey samples collected at a different time point (36, 42, 57 and 81 hours) from a cow with *Streptococcus uberis* infection (Mudaliar, et al., 2016).


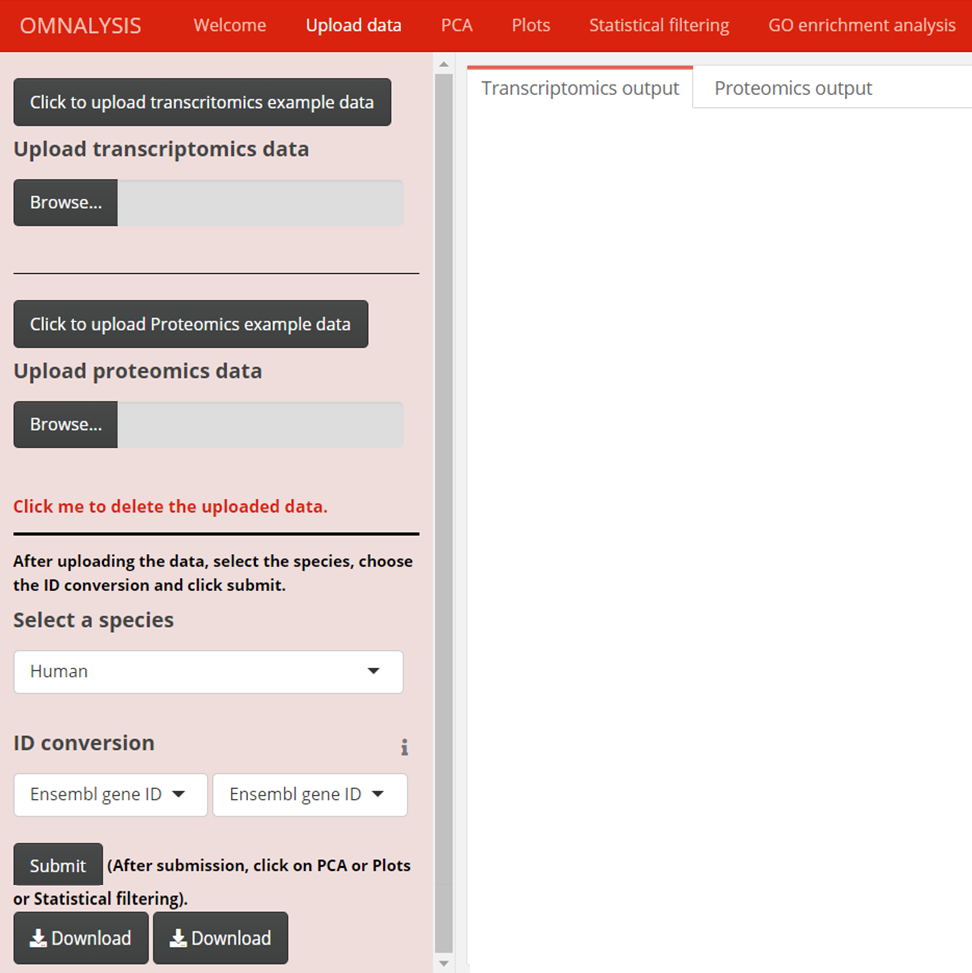


1. Select the **Upload transcriptomics data Browse tab,** to upload your transcriptomics data in CSV file up to four experimental conditions with expression value (log fold change), a measure of significance (*P* value), count of reads (log counts per million).
2. Select the second **Upload proteomics data Browse tab,** to upload your proteomics data in xlsx format file up to four experimental conditions with UniProt ID, FDR-adjusted P-value and Fold Change.
3. To delete the uploaded file using the red color ‘Click me to delete the uploaded data.’.
4. Once the data is loaded (**ONLY ONE OMICS TYPE at a time**) on the window panel, select the species provided in the **select a species tab** (Human, Chicken, Pig, Cow, Mouse, Rat, Dog, *Drosophila melanogaster* and *C. elegance*) with the help of a dropdown menu to perform further analysis.
5. Gene ID conversion is mandatory for further analysis. The converted IDs will be used for the volcano PCA and Volcano plots.
6. After selecting the species user needs to convert the less informative ENSEMBLGENE ID or UniProt ID to more informative IDs using the **ID conversion drop-down menu tab**.
7. Press the **SUBMIT button** to perform the ID conversion.
8. In the **Transcriptomics or Proteomics output** window, the converted IDs will be added to the last column of the uploaded data.
9. Press the first **Download button** to download the converted ID table of transcriptomics and the Second **Download button** for proteomics converted IDs in CSV format.

# PCA

1. Principal component analysis (PCA) is an unsupervised dimension reduction method that allows us to understand the relationships among the attributes of expression data. PCA analysis calculates the principal components using the Euclidean distance and linear transformation of the expression data. It calculates the most significant variable in the provided data to calculates the first principal component (PC1), second principal component (PC2) and so on.


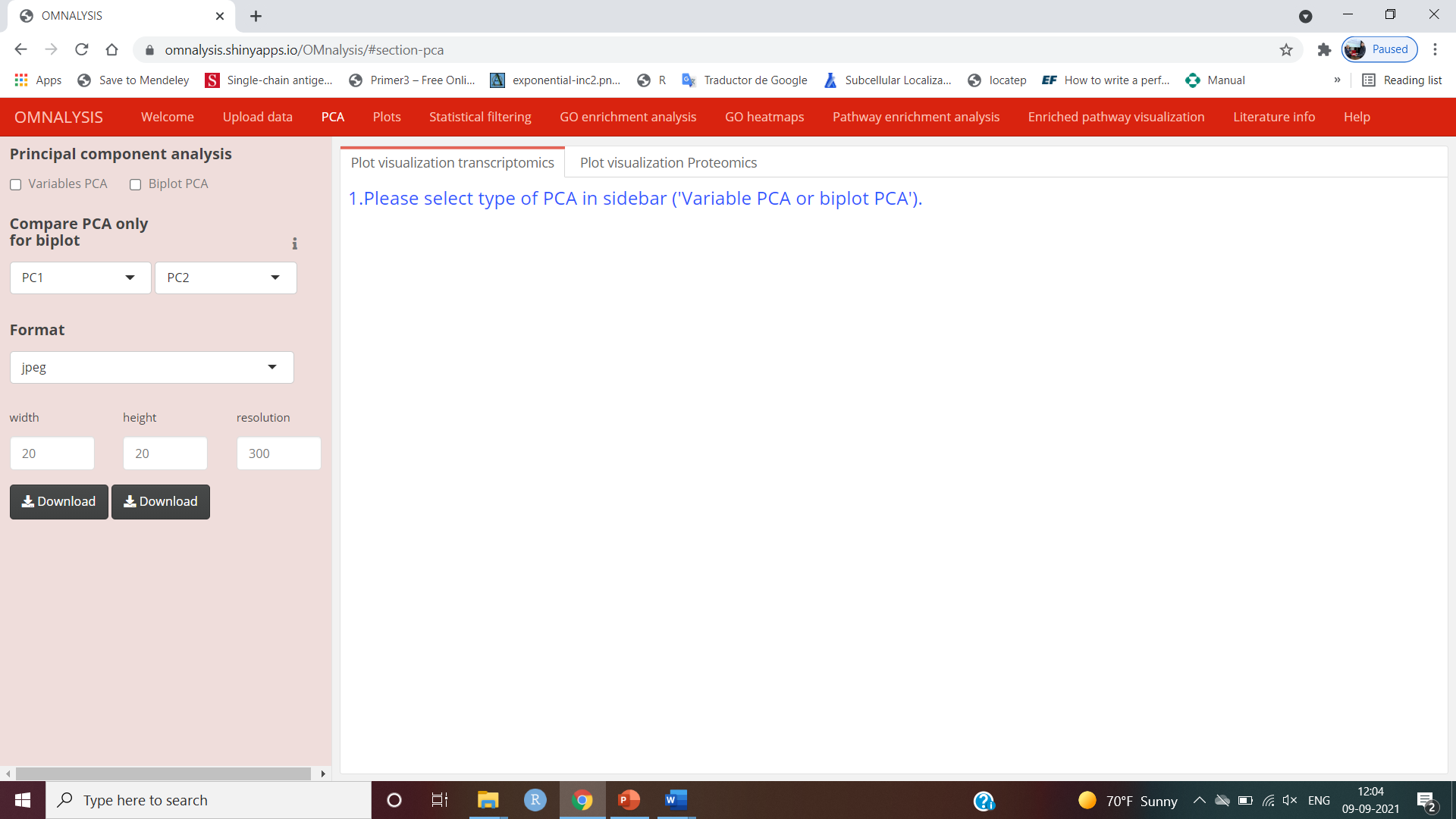


1. Two types of PCA plots are provided, first is a **Variable PCA** plot that provides information about the direction and relationship among the variables (treatments) and the second is **Biplot PCA** that visualize the features of variable PCA plot and observation (genes or proteins) of each treatment in a single plot.
2. Press **Variable PCA or Biplot PCA** to generate transcriptomics variable PCA plot, biplot PCA plot in the **Plot visualization transcriptomics window**.
3. Press **Variable PCA or Biplot PCA** to generate proteomics variable PCA plot, biplot PCA plot in **Plot visualization proteomic window.**
4. Most of the variables are explained in PC1 and PC2, however, to compare principal components, select PC from **Compare PCA for Biplot only drop-down tab** is to **explore and compare the other available PCs (PC1 vs PC2, PC2 vs PC3 and PC3 vs PC4)**.
5. Use **Format drop-down menu** to download images in **jpeg, png, pdf and tiff** formats.
6. Provide **width, height** and **resolution** to the **output PCA plot** using **numerical input tabs**. The default values are **20cm, 20cm and 300px for width, height and resolution** respectively.
7. Press the **first Download button** to download transcriptomics **PCA plots** and the **second Download button** to download proteomics **PCA plots.**

# Plots

1. Scatter plot uses log fold change versus log counts per million to visualize your expression data in the form of up and down-regulated genes. Each dot in the scatter plot represents up-regulated genes (green colour), non-significant genes (black colour) and downregulated genes (red colour).


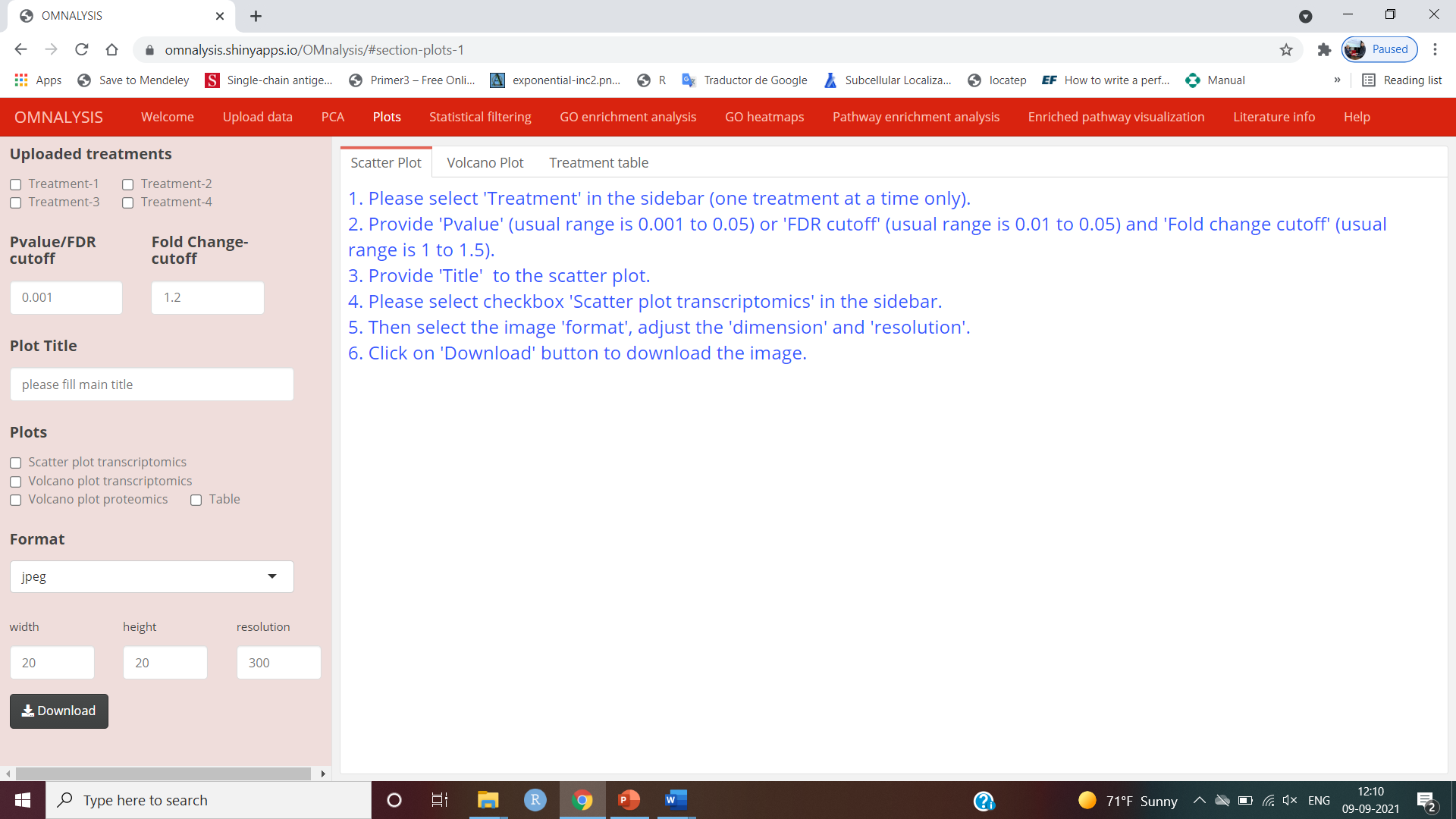


1. Select from the **Uploaded treatments** checkboxes, Example- Treatment-1, then provide the **numeric input Pvalue/FDRcutoff** for both scatter and volcano plot and **Fold Change-cutoff for volcano plot** to generate plots. The default numeric **Pvalue cutoff** is 0.001 and **Fold Change-cutoff** is 1.2 for transcriptomics and can be changed.
2. Provide plot name (Example- “Scatter plot of hbmec induced with WNV”) in **Plot Title text input option**.
3. Press one of the checkboxes in the **Plots heading (one at a time only)** to generate a type of plot and **Table** to visualize each treatment data with converted ids. To display the results click on the windows named **Scatter plot, Volcano plot and Treatment table**.
4. Select image format type from the **Format** drop-down menu.
5. Provide **width, height** and **resolution** to the **output scatter and volcano plots** using **numerical input tabs**. The default values are **20cm, 20cm and 300px for width, height and resolution** respectively.
6. Press the **Download button** to download the **scatter or volcano plots.**

# Statistical filtering


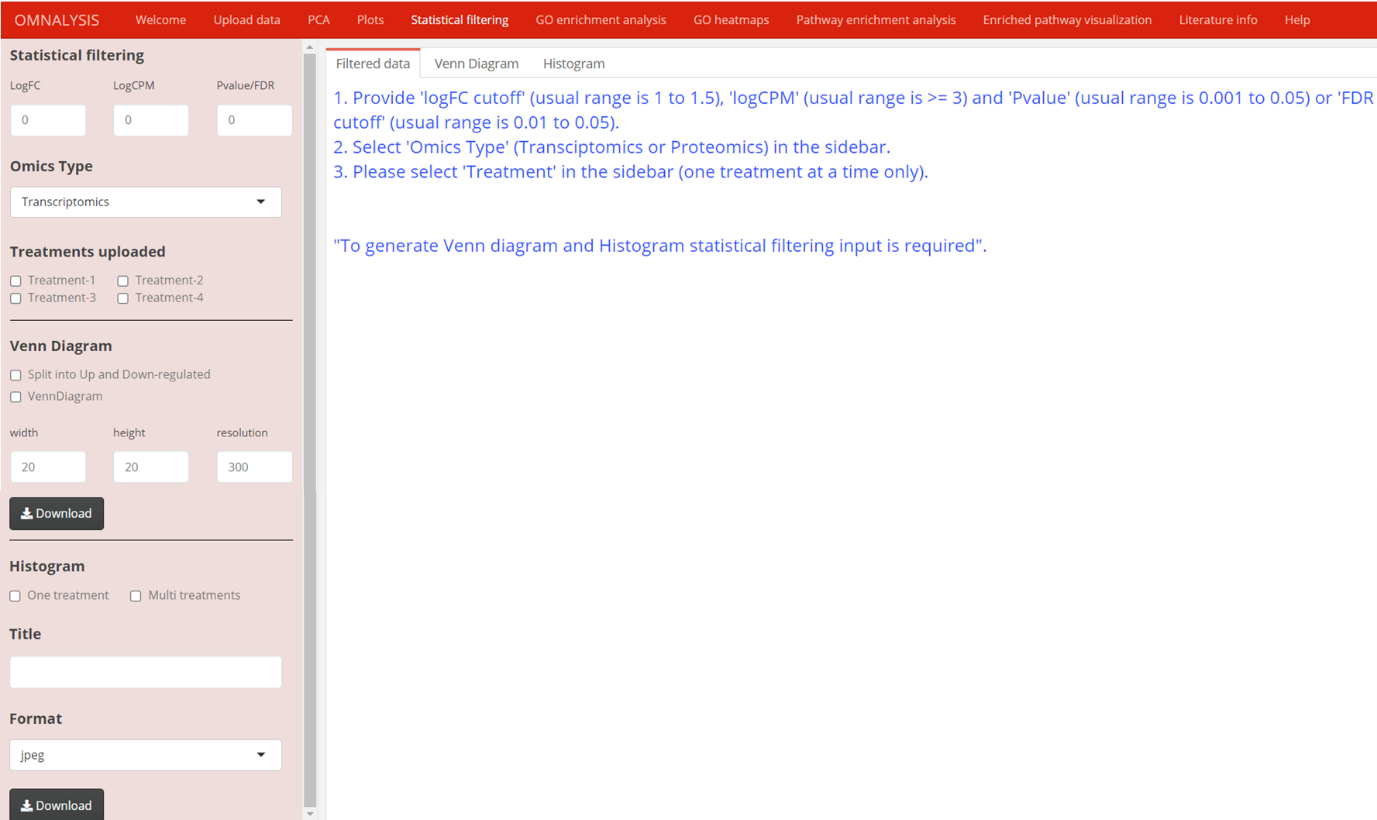


1. Provide threshold numerical values in **the Statistical filtering tab input boxes** (**LogFC, LogCPM, Pvalue**) provided to filter out the genes that are unable to cross the threshold values. The default value is 0 for all components.
2. Select one from the **Omics Type** drop-down tab (**Transcriptomics or Proteomics**).
3. Use checkboxes provided under **Treatments** **uploaded** header **(Treatment-1, Treatment-2, Treatment-3 and Treatment-4)** to visualize the data, Venn diagram and histogram of the selected treatments on **Filter data, Venn Diagram and Histogram** display panel.
4. **Statistical filtering is mandatory for the Venn diagram, histogram and further analysis.**
5. Once the filtered data is visible on the **Filter data panel.** Use the checkboxes provided under the **Venn Diagram header** to visualize the common significantly DEGs or abundance protein in the uploaded treatments. Use **Split into Up and Down-regulated checkbox** to obtain common or different up and down-regulated genes in two groups of uploaded treatments. Use the **VennDiagram checkbox** to obtain common genes in the selected treatments.
6. Provide **width, height** and **resolution** to the **output Venn diagram and Histogram** using **numerical input tabs**. The default values are **20cm, 20cm and 300px for width, height and resolution,** respectively.
7. Use first  **Download tabs,** to download the Venn diagram and second to download the histogram.
8. Use checkboxes provided below the **Histogram header**. Click on the **One treatment checkbox** to generate each treatment histogram showing the number of differentially expressed genes or proteins that falls on the log fold change range or click on the **Multi treatments checkbox** to generate the total number of up and down-regulated genes or proteins available in each treatment in a single histogram.
9. Use the **Title** input tab to provide the title to the generated Venn diagram or histogram (example: - “Venn diagram or Histogram of Treatment-1”)
10. Select **image format types using the drop-down Format option.**

# Gene ontology (GO) enrichment analysis

1. Select the **Omics Type** according to the uploaded data.
2. Use the checkboxes under the **Gene ontology classes header** to perform the categorical analysis of your uploaded data.
3. Clicking on the **GO Biological Process (BP)**-multiple molecular activities integrate to perform a process or **GO Molecular Function (MF)**-activities at the molecular level by gene product or **GO Cellular Component (CC)**-site of function concerning cellular structure.


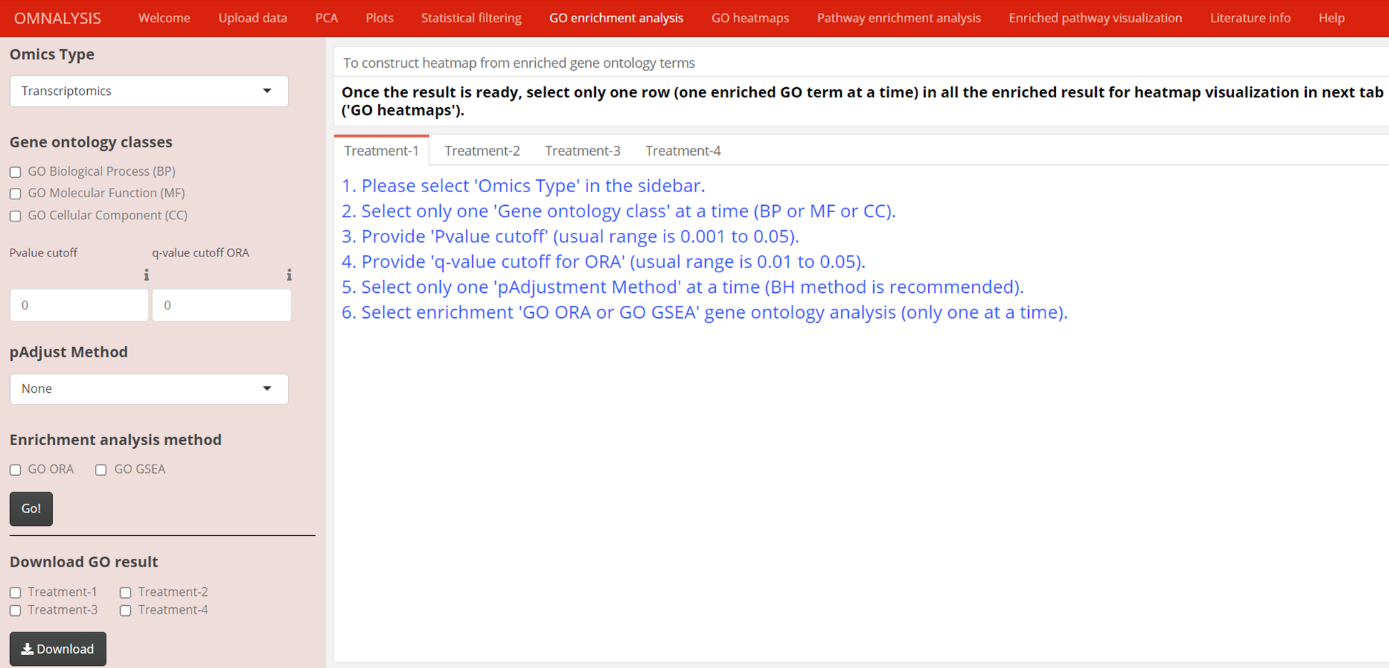


1. After selecting one of the **Gene ontology classes,** provide **Pvalue cutoff** and the **q-value cutoff ORA**. The default Pvalue and q-value cutoff is 0 and can be changed.
2. Select one of the p-value adjustment methods from **pAadjust Method drop-down menu**. List are **Holm, Hochberg, Hommel, Bonferroni, Benjamini and Hochberg (BH), Benjamini and Yekutieli (BY)** and **FDR** to control false positive results in the analysis. The first four methods control the family-wise error rate (probability of making one or more false discoveries) and the remaining methods control the expected proportion of discoveries that are rejected falsely (FDR). **We suggest FDR correction methods for a more reliable result**.
3. Select one of the provided **Enrichment analysis methods, GO ORA** (based on hypergeometric test and mapping of genes to the annotated biological vocabulary) or  **GO GSEA** (based on the Kolmogorov Smirnov test and consider gene set with their sorted log fold change value).
4. After providing the necessary inputs mentioned above, click on the **Go! button** to launch the enrichment analysis. **Please keep in mind that the same input values will be used for all treatments to maintain the standard of enrichment analysis**.
5. The result of the enrichment analysis is visualized on the  **Treatment-1, Treatment -2, Treatment -3, and Treatment -4**  display panels.
6. From **the ontology result table**, **select one enriched GO term by selecting only one row and the same GO term in all the treatments**. The selected information will be used for the **generation of heatmaps in the next GO heatmaps section**.
7. Use checkboxes provided under the **Download GO result header** to download the result in CSV format.
8. Select one **Treatment checkbox** at a time and click on the **Download button**.

# GO heatmaps

1. GO heatmaps section of OMnalysis provide visualization of the enrichment analysis using heatmaps (expression values of genes that mapped to the biological term in gene ontology enrichment analysis) and word cloud.


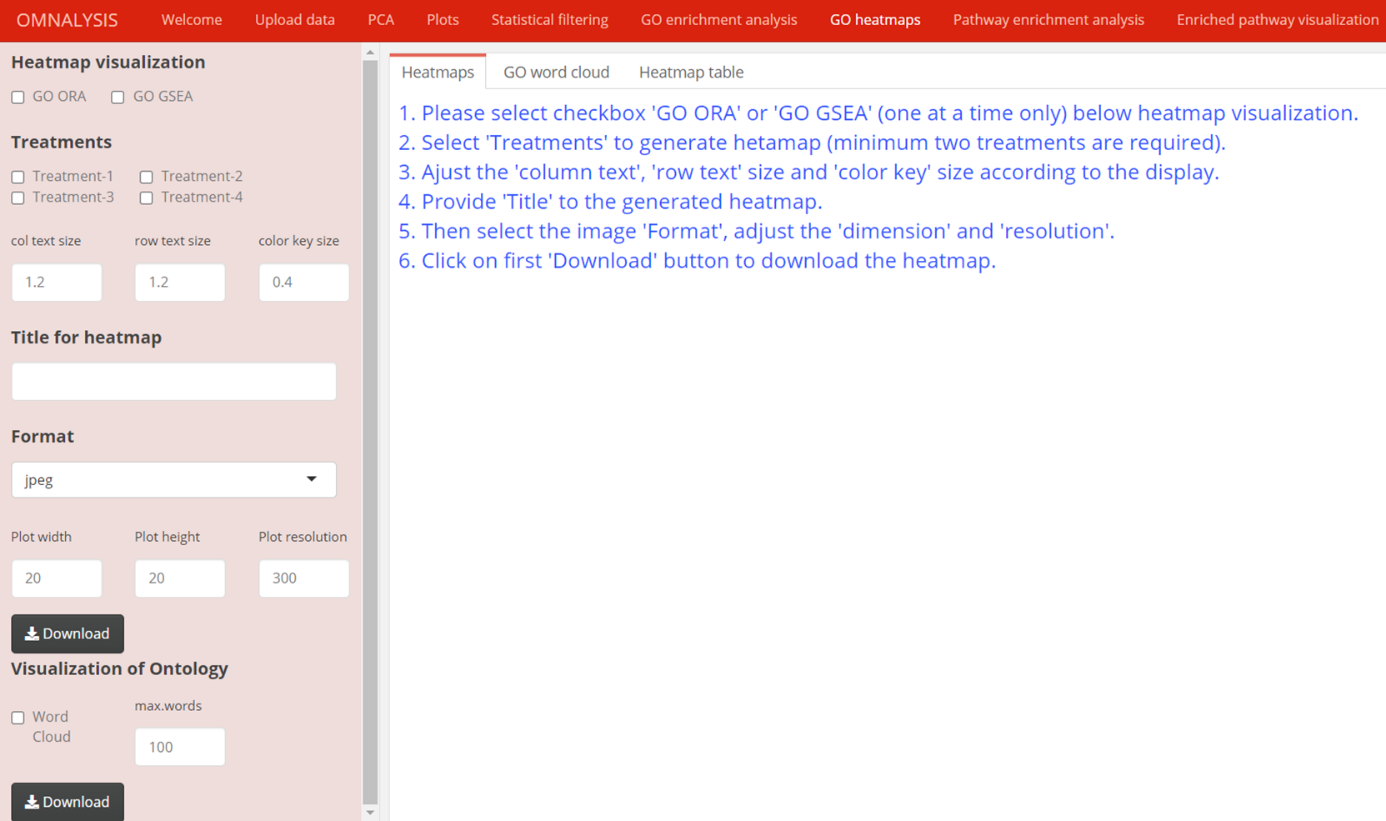


1. It is mandatory to **select at least two enriched GO terms (rows) from the previous tabular output of GO enrichment analysis.**
2. Select one of the **GO ORA** or **GO GSEA** methods from **Heatmap visualization**.
3. Click on at least two checkboxes **(Treatments 1, 2, 3 and 4)** provided under the **Treatments header** to generate a **heatmap.**
4. Adjust the column text, row text and color key size using **col text size, row text size** and **col key size numerical input tabs, respectively.** The default value for **col text size, row text size** and **col key size** are **1.2, 1.2 and 0.04,** respectively.
5. Input text in the **Title for heatmap input tab** to provide main text on the heatmap.
6. Download **Heatmap and word cloud** using the image formats provided under the **Format drop-down menu.**
7. Adjust **dimension and resolution of heatmap and word cloud** using **numerical input in tabs Plot width, Plot height and Plot resolution**. The default value is 20cm, 20cm and 300px for width, height and resolution, respectively.
8. After providing the above-mentioned information **download the generated heatmap using the first Download button.**
9. Click on the **Word cloud** checkbox of the selected treatment provided under the **Visualization of Ontology header.**
10. Provide the numeric input (**maximum number of words you want to visualize in the word cloud) using max.words input tab option**. The default value of the maximum words in the **word cloud** is 100.
11. Press the second **Download button** to download the generated word cloud diagram.

# Pathway enrichment analysis

1. This section provides the databases and methods available to perform pathway analysis.


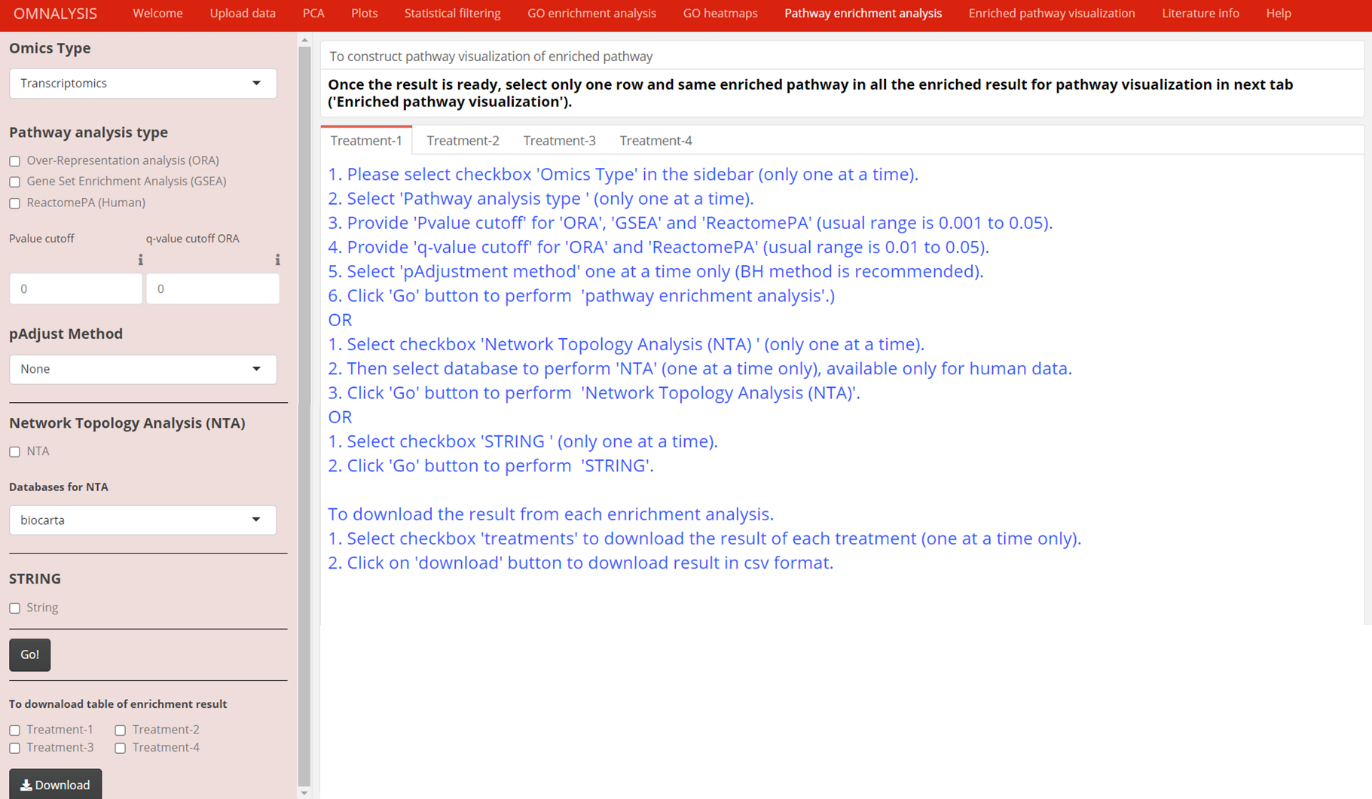


1. Select the **Omics Type** according to the uploaded data.
2. Select one of the options provided in **Pathway analysis type** **(**O**ver-represented analysis (ORA), Gene set enrichment analysis (GSEA), ReactomePA (Human))** to perform pathway enrichment analysis.
3. Provide numeric input in the **Pvalue cutoff tab** and select one of the **pAdjusted Method tabs** to overcome the chances of getting a false-positive result. Note that stringency cutoff value may result in a fewer number of gene set enrichment pathways.
4. If using O**ver-represented analysis (ORA)** provide q-value cutoff value in **q-value cutoff ORA numeric input tab**.
5. P-value and the q-value cutoff is applicable only for methods under **Pathway analysis type**.
6. To perform **Network Topology Analysis (NTA)** use the **NTA checkbox** provided under the **Network Topology Analysis (NTA)**. This method is supported by **four database**s first, **biocarta (protein sets participating in the pathway),** second, **panther (a curated and comprehensive database to classify protein and their genes through evolutionary relationship),** third, **NCI-Nature Pathway Interaction Database (Signaling pathways composed of human biomolecular interactions and cellular processes) and** fourth, **pharmgkb (a comprehensive resource that provides information about how human genetic variation affects the response to medications).**
7. To perform STRING enrichment analysis, click on the **String checkbox** provided under the **STRING header**.
8. Once the above steps are checked, and anyone pathway enrichment analysis was selected, click on the **Go! button** to launch the pathway enrichment analysis.
9. Click on the  **“Treatment-2, Treatment-3, and Treatment -4" display panels to perform pathway enrichment analysis for each treatment.**
10. From the pathway enrichment result table, **select only one row with the same enriched pathway in all treatments**.
11. The selected row or enriched pathway information will be used for the visualization of the expressed gene or proteins in the next section **Enriched pathway visualization**.
12. Click on the **Treatments checkboxes one at a time only** provided under the **To download table of enrichment result** **header**.
13. Click on the **Download button** to download the result in **CSV format**.

# Enriched pathway visualization

1. After selecting the single and same pathway in all the treatments from the **pathway enrichment result table**, you can visualize the pathway using the checkboxes provided under the **Pathway visualization header and according to the pathway enrichment method performed in the Pathway enrichment analysis section of OMnalysis.**


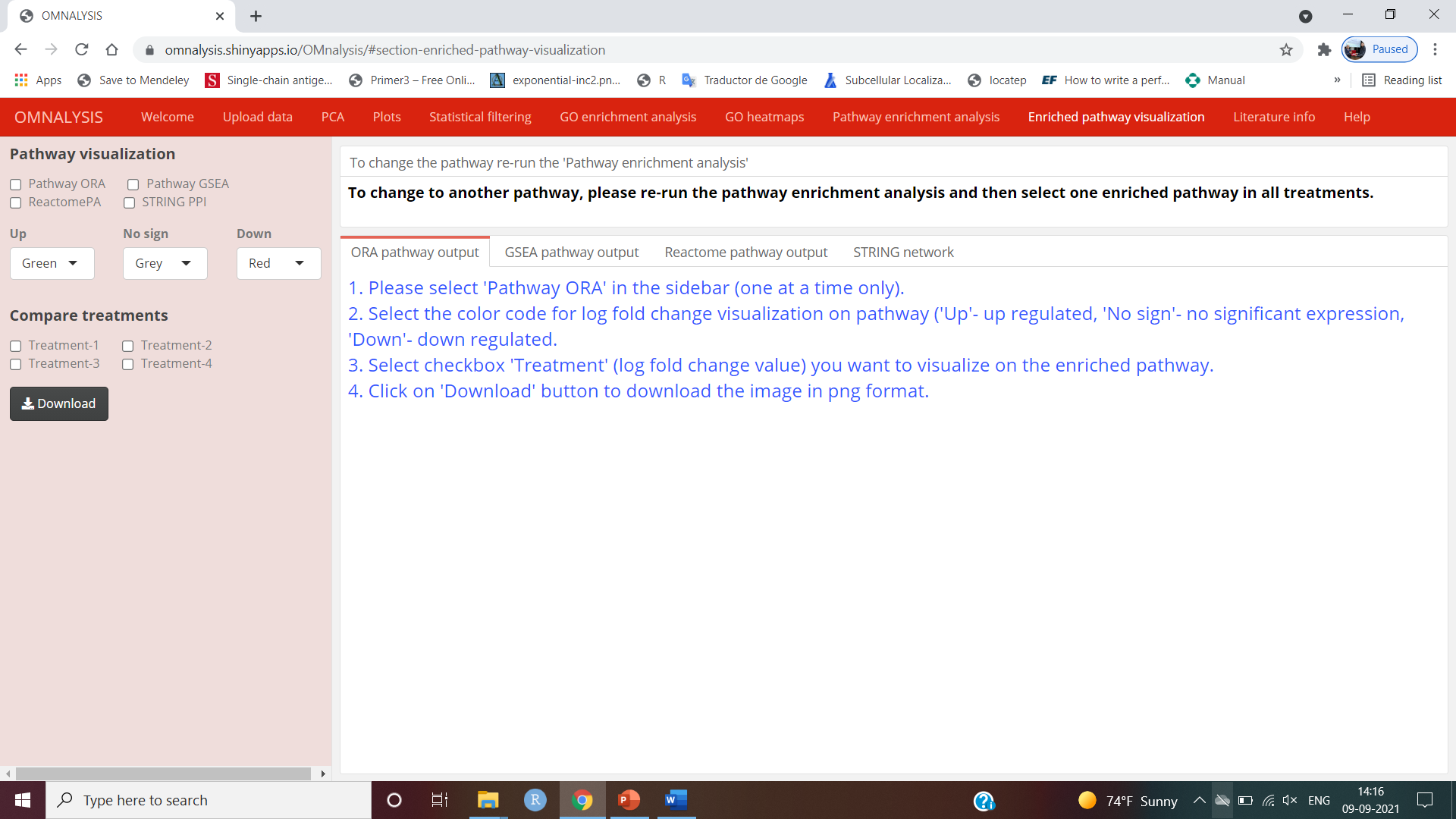


1. Select one of the **Pathway ORA, Pathway GSEA, ReactomePA** and **STRING PPI** checkboxes provided under the **Pathway visualization header**.
2. If the previous pathway enrichment analysis was ORA, then only the ORA enriched pathway will be visualized in the **ORA pathway output subtab**, **if not, it will show an error**.
3. Select the color code from three drop-down tabs for **highly expressed (Up), No induced or absent expressed (No sign) and suppressed genes or proteins (Down).**
4. The default colour code for **Up** is green, **No sign** grey and **Down** in red. You can **select more colour combinations from the none, red, green, yellow, blue, and grey to visualize the expression values (logFC) on the enriched pathway.**
5. Select the **Treatments checkboxes provided under the Compare treatments header** to visualize the enriched pathway **of Treatment-1, Treatment-2, Treatment-3, Treatment-4.**
6. Click on **ORA pathway output or GSEA pathway output or Reactome pathway output or STRING network display panel, according to the enriched pathway method selected.**
7. The output from the **Reactome pathway analysis** can be interpreted using the below notation image.

[
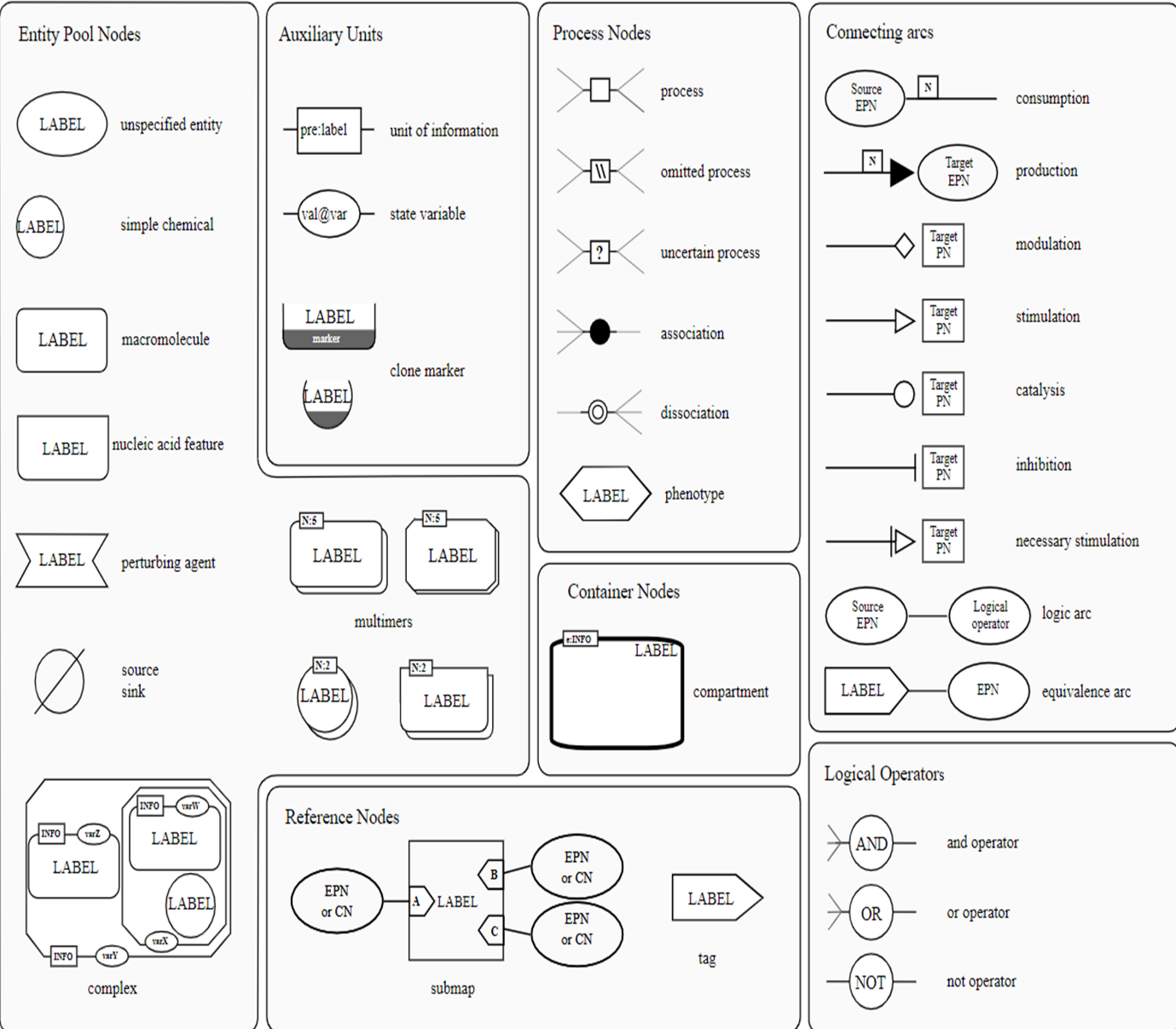
](https://github.com/Punit201016/OMnalysis/blob/main/www/om_10.PNG)

1. Keep in mind that the visualization of pathways depends on the **pathway enrichment analysis method and selection of the same pathway in all treatments.**
2. Click on the **Download button** to download the **pathway image of ORA, GSEA and ReactomePA and STRING output in PNG image format**.


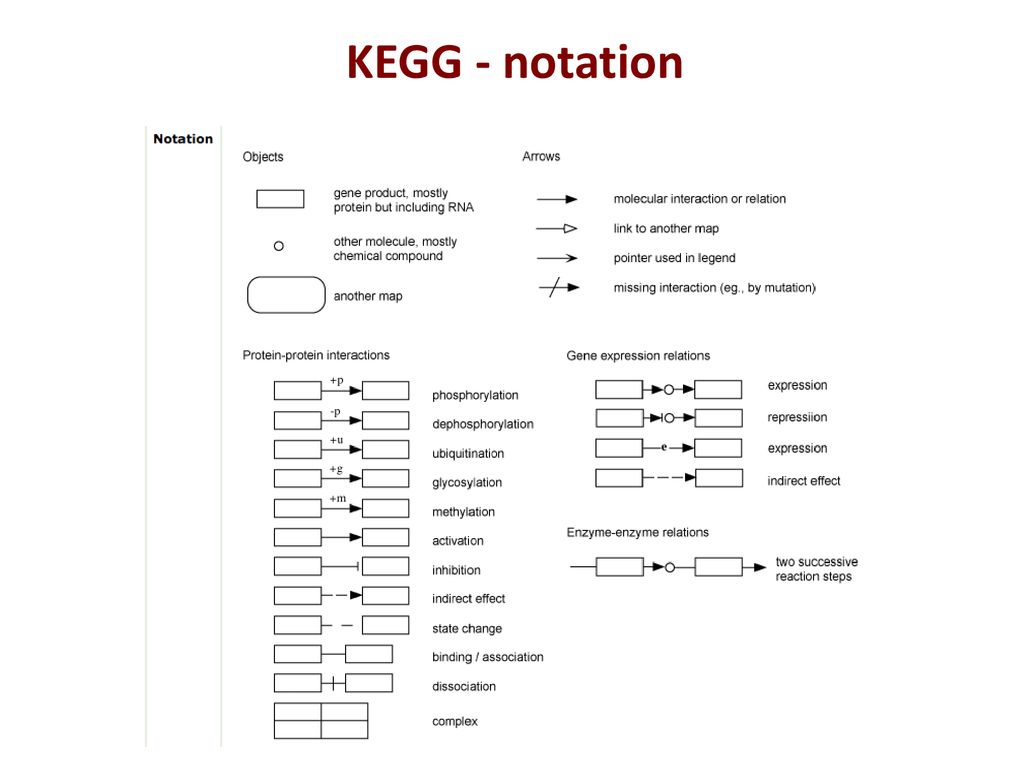


# Literature info


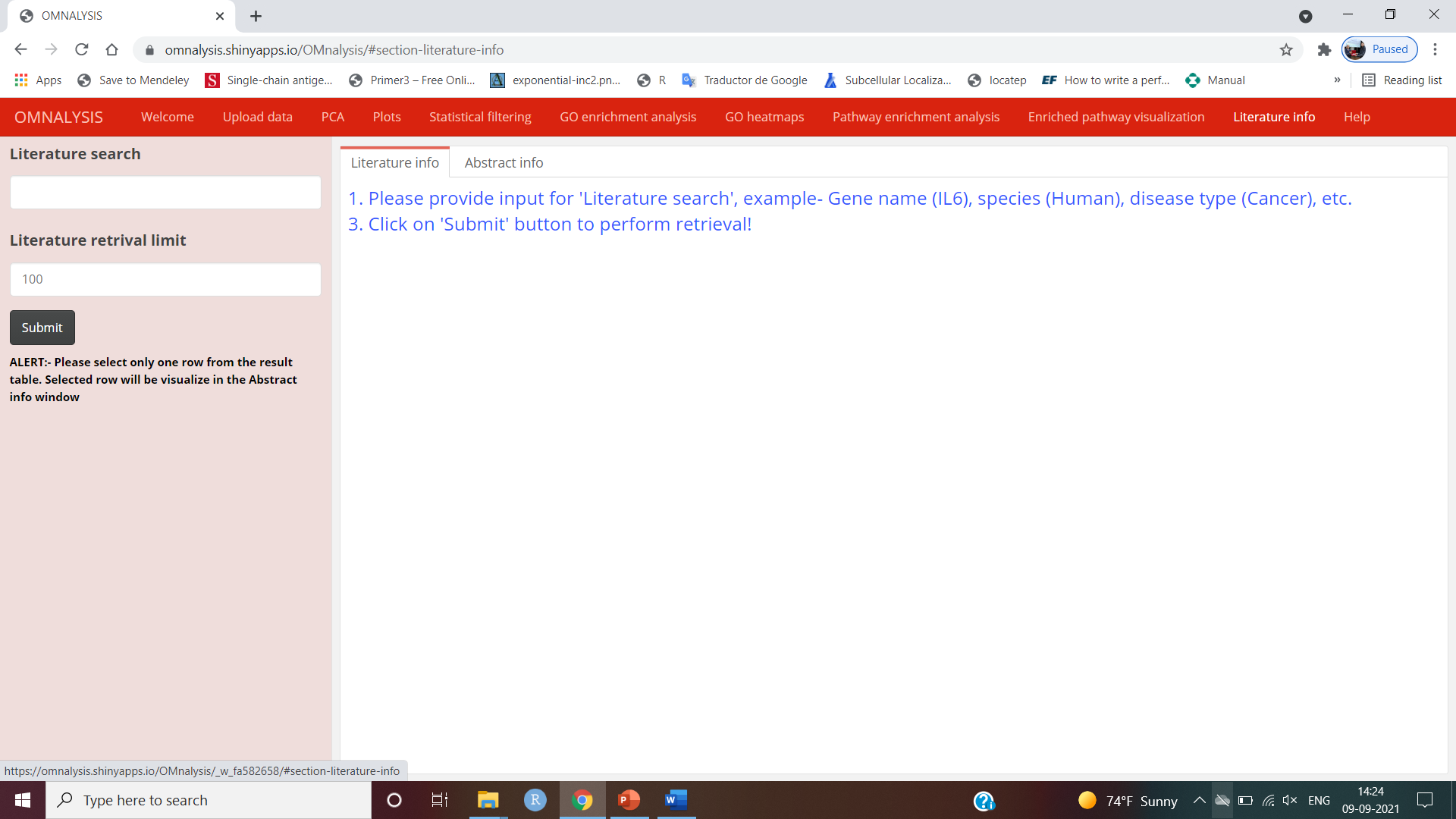


1. This section provides the option to retrieve the information from the Europe PMC by providing **biomarkers name, species, disease, cell or tissue type** in the **text input tab below Literature search.**
2. The **Literature retrieval limit** has an input option to provide a number that will decide the fetching of the literature. The default is 100.
3. Once the literature search and retrieval limit are provided, you can **proceed with the submit button to perform the retrieval of scientific literature.**
4. The result in tabular form will appear in the **literature info subtabs**.
5. **Select one scientific literature row** in the **literature info table** at a time to view the abstract and other information on the **next subtab Abstract info.**
6. If the keyword provided in the Literature search is not correct then the result may produce an error or blank table of literature info.
